# The efficacy of azithromycin combined with seven types of Chinese medicine injections in the treatment of *Mycoplasma pneumoniae* pneumonia in children: a systematic review and Bayesian network meta-analysis

**DOI:** 10.3389/fphar.2024.1378445

**Published:** 2024-09-24

**Authors:** Xinggui Huang, Sian Tao, Chenhao Liu, Xiaoluo Sun, Yule Hao, Yuqi Ma, Yi Liu, Jibin Liu

**Affiliations:** School of Basic Medical Sciences, Chengdu University of Traditional Chinese Medicine, Chengdu, China

**Keywords:** azithromycin, *Mycoplasma pneumoniae* pneumonia, antibiotic resistance, Bayesian network meta-analysis, Chinese medicine injections

## Abstract

*Mycoplasma pneumoniae* pneumonia (MPP) is the predominant community-acquired pneumonia (CAP) in children aged 5 years or older. In recent decades, the annual increase in drug resistance rates of macrolide antibiotics, particularly azithromycin (AZ), has led to complex clinical treatment strategies and substantial healthcare costs associated with MPP. Chinese medicine injections (CMIs), recognized as an effective supplementary therapy, are acknowledged by clinicians in China. It is necessary to explore the efficacy of azithromycin in combination with CMIs.

**Methods:**

Randomized controlled trials (RCTs) evaluating azithromycin in combination with seven types of CMIs for MPP in children were identified based on inclusion criteria and assessed using the revised Cochrane risk of bias tool (RoB 2.0). R 4.3.1 and STATA 15.0 were employed to generate ranking probabilities and perform network meta-analysis. Competing interventions were ranked using the surface under the cumulative ranking (SUCRA) probabilities.

**Results:**

A comprehensive analysis was performed on 155 RCTs involving 15,014 patients and 8 therapeutic strategies within this Bayesian network meta-analysis (BNMA). The results indicated that AZ combined with seven types of CMIs was more effective than azithromycin alone in overall outcomes. Notably, azithromycin combined with Chuanhuning injection (AZ + CHN) achieved the highest ranking in improving the clinical effectiveness rate (SUCRA, 80.89%); regarding secondary outcome measures, azithromycin combined with Yanhuning injection (AZ + YHN) had the highest probability of improving four different outcomes: disappearance time of cough (SUCRA, 80.01%), disappearance time of pulmonary rale (SUCRA, 87.77%), disappearance time of fever (SUCRA, 95.70%), and disappearance time of pulmonary shadows in *X*-ray (SUCRA, 97.34%); furthermore, azithromycin combined with Qingkailing injection (AZ + QKL) was more likely to reduce average hospitalization time (SUCRA, 94.60%).

**Conclusion:**

This study highlights the potential benefits of seven types of Chinese medicine injections as adjunctive therapy for *Mycoplasma pneumoniae* pneumonia in children. However, further support and validation of these findings are needed through high-quality randomized controlled trials with larger sample sizes and double-blind designs.

**Systematic Review Registration:**

https://www.crd.york.ac.uk/PROSPERO/#recordDetails/.

## Introduction

*Mycoplasma pneumoniae* is a unique bacterial pathogen associated with a diverse range of clinical manifestations, encompassing pneumonia, upper and lower respiratory infections, and extrapulmonary manifestations (Waites and Talkington, 2004; Sánchez-Vargas and Gómez-Duarte, 2008; Narita, 2016). *Mycoplasma pneumoniae* pneumonia (MPP), recognized globally as a prevalent cause of community-acquired pneumonia (CAP), accounts for up to 40 percent or more of CAP cases (Waites et al., 2017; Shah, 2019). Macrolides, especially azithromycin (AZ), have been the first-line treatment recommended for several decades (Kawai et al., 2013; China, 2023). However, with the extensive application of macrolide antibiotics, new characteristics of pediatric MPP have been presented. First of all, the emergence of *macrolide-resistant mycoplasma pneumoniae (MRMP)* challenges empiric macrolide therapy (Vester and Douthwaite, 2001; Wang et al., 2012). Since *MRMP* was first reported in Japan in 2000, the prevalence of macrolide-unresponsive MPP has steadily increased over the past 2 decades (Hong et al., 2013; Kim et al., 2022), creating a therapeutic conundrum, particularly for young children, in whom tetracyclines or fluoroquinolones are relatively contraindicated (Lee et al., 2018). Furthermore, some children with MPP who are treated with macrolides for 7 days or more may still exhibit persistent fever, clinical signs, worsening lung imaging findings, and extrapulmonary complications (China, 2023). Additionally, there has been a gradual increase in the incidence of MPP in children, resulting in elevated medical care costs and increased socioeconomic burdens (Kutty et al., 2019; Beeton et al., 2020). MPP has garnered widespread concern among pediatricians.

In traditional Chinese medicine theory, MPP is categorized under “pneumonia and asthma” and “external fever,” with the primary treatment approach focused on clearing away heat and toxic materials. Research has demonstrated that botanical drug decoctions with heat-clearing and detoxifying properties, such as Wuhu Decoction, are beneficial for patients with MPP (Yang et al., 2024). With the outbreak of COVID-19, guidelines have incorporated heat-clearing and detoxifying Chinese medicine injections (CMIs) for treating severe and critically ill patients (Wang X. Y., 2020; Wang et al., 2020). The efficacy and characteristics of these treatments have gradually drawn extensive worldwide attention. Relevant studies have reported that heat-clearing and detoxifying CMIs can safely and effectively reduce clinical symptoms, lower the case fatality rate, and decrease the conversion rate of critically ill patients (Chun et al., 2021; Zhang X. Y. et al., 2021). Currently, heat-clearing and detoxifying CMIs are widely applied in lung diseases, with randomized controlled trials confirming their pivotal role in improving clinical symptoms, impeding disease progression, and minimizing adverse reactions (Niu et al., 2021; Yang et al., 2022; Wei et al., 2023).

All seven injections selected for this study were developed based on heat-clearing and detoxifying principles and were authorized by the China Food and Drug Administration (CFDA). Given the varying efficacy of different injections, this study aims to evaluate the clinical efficacy of azithromycin combined with various CMIs, contributing more evidence for informed selection.

## Materials and methods

To enhance the accuracy and reproducibility of the study, CMIs were reported in accordance with The ConPhyMP consensus (Heinrich et al., 2022); the results are shown in Supplementary Materials S1, S2. We standardized the scientific names of botanical drugs and validated them in the “Plants of the World Online” database. Summary tables detailing the composition of CMIs and their reporting in the original study were prepared in accordance with the principles outlined in the four pillars of ethnopharmacology. The composition and standard name of each injection are provided in Table 1, and other details are provided in Supplementary Material S3.

**TABLE 1:** Composition and standard names of seven CMIs.

Drug name	Botanical plant name	Species	Parts and form used
Reduning injection	*Artemisia annua L.*	Asteraceae	Dried aerial parts
	*Lonicera japonica* Thunb	Caprifoliaceae	Dried stem and branch
	*Gardenia jasminoides J.* Ellis	Rubiaceae	Dried ripe fruit
Tanreqing injection	*Scutellaria baicalensis* Georgi	Lamiaceae	Dried root
	*Forsythia suspensa (Thunb.)* Vahl	Oleaceae	Dried fruit
	*Lonicera japonica* Thunb	Caprifoliaceae	Dried stem and branch
Xixinnao injection	*Acorus calamus* var. *angustatus* Besser	Acoraceae	Dried rhizome
Qingkailing injection	*Gardenia jasminoides J.* Ellis	Rubiaceae	Dried ripe fruit
	*Scutellaria baicalensis* Georgi	Lamiaceae	Dried root
	*Lonicera japonica* Thunb	Caprifoliaceae	Dried stem and branch
	Isatidis Radix	Brassicaceae	Dry leaf
Chuanhuning injection	*Potassium dehydroandrographolide succinate*	-	Dried aerial parts
Xiyanping injection	*Andrographolide sulfonate*	-	Dried aerial parts
Yanhuning injection	*Potassium sodium dehydroandrographolide succinate*	-	Dried aerial parts

This BNMA was registered with PROSPERO (CRD42023425176), which follows the Cochrane Handbook criteria and Preferred Reporting Items for Systematic Reviews and Meta-Analyses: PRISMA statement (Hutton et al., 2015).

### Data sources and search strategy

We conducted a comprehensive search across eight databases: China National Knowledge Infrastructure (CNKI), Wanfang Database, Database of Chinese Sci-tech Periodicals (VIP), Chinese Biomedical Literature Database (CBM), PubMed, Cochrane Library, Embase, and Web of Science, from their inception to 24 May 2023. Both MeSH terms and free words were used to retrieve relevant randomized controlled trials (RCTs). The search strategies are detailed in Supplementary Material S4.

### Inclusion criteria

Clinical trials meeting the following criteria were eligible for inclusion: (1) study type: the study type was distinctly referred as a clinical RCT in the literature; (2) subjects: patients under 18 years of age with a definitive diagnosis of *Mycoplasma pneumoniae* pneumonia (China, 2023); (3) intervention measures: studies using azithromycin alone as the control group, while the observation group received one of the azithromycin combinations for the treatment of MPP, with controlled confounding factors; and (4) evaluation indexes: the primary outcome indicator was the clinical effectiveness rate, with secondary outcome indicators including disappearance time of cough, disappearance time of pulmonary rale, disappearance time of fever, average hospitalization time, disappearance time of pulmonary shadows in *X*-ray, and adverse reaction reports.

### Exclusion criteria

Exclusion criteria were as follows: (1) studies that did not specify being a “randomized controlled trial” or “RCT”; (2) children who received other joint interventions such as acupuncture, cupping, moxibustion, massage, or acupoint application; (3) studies with unclear outcome indicators or incomplete data; and (4) research types that were reviews, theoretical discussions, summaries of experience, case reports, and animal-based experiments.

### Data extraction

Two researchers (XGH and XLS) extracted information using a pre-designed extraction form. From each study, the collected data are (1) study characteristics, including the name of the first author and the publication year; (2) participant information, such as the sample size, gender, age, and course of disease; (3) details of interventions, dosage, and duration; and (4) outcome measures. Discrepancies between the two researchers in the process of study selection were resolved by consensus or negotiation with a third researcher (SAT).

### Quality assessment

The Cochrane risk of bias tool 2.0 (RoB 2.0) (Sterne et al., 2019) was adopted to evaluate the methodological quality of selected studies based on the considerations below. RoB 2.0 assesses the risk of bias from five domains, namely, the bias generated in the random process, bias deviating from the established intervention, bias of missing outcome data, bias of outcome measurement, and bias of selective reporting of results. Two independent researchers (YLH and XLS) conducted the RoB 2.0 evaluation, and any discrepancies were resolved by a third reviewer (YQM).

### Statistical analysis

Statistical analysis was performed using R 4.3.1 software and STATA 15.0 software. The clinical effectiveness rate was analyzed using the odds ratio (OR) with 95% confidence intervals (CIs). Due to the nature of the dichotomous variables, the terms “cured,” “effective,” and “significantly effective” of the clinical effectiveness rate from the included literature studies were all regarded as “effective,” and mean differences (MDs) with 95% CIs were used to analyze other outcomes. The funnel plot was drawn and compared to determine whether publication bias existed in this network meta-analysis using STATA 15.0 software. The network evidence map was drawn by STATA 15.0 software to show the direct and indirect comparisons between different interventions. A BNMA was conducted using R software 4.3.1 *via* the gemtc package. The models were optimized using the Markov chain Monte Carlo (MCMC) method with a weighted sample size running in four chains. The number of iterations was set to at least 60,000 to obtain model convergence. Convergence was assessed by the Brooks–Gelman–Rubin diagnosis plot and potential scale reduction factor (PSRF), with a PSRF value close to 1, indicating convergence. Since there were no “closed loops” in the network plot, we were unable to assess inconsistency among direct and indirect comparisons, adopted a consistency model for analysis, and a random- or a fixed-effect model was used based on the results of DIC and *I* ^*2*^. The CMIs were compared using the surface under the ranking (SUCRA) plot; the SUCRA curves indicate the most effective and least effective treatments in percentages of 100% and 0%, respectively.

## Results

### Search results

**FIGURE 1:**
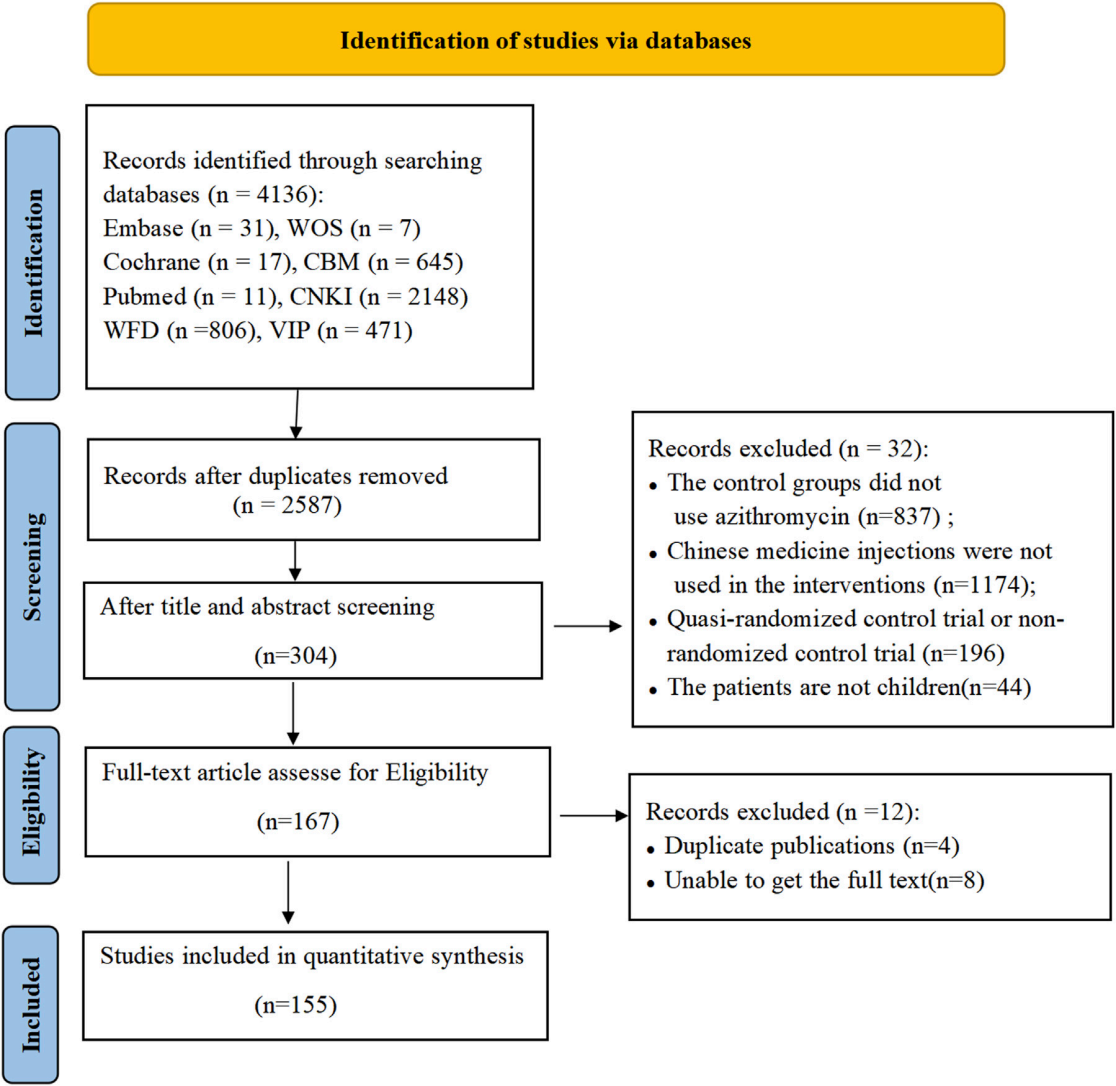
Identification of studies via databases.

A total of 4,136 publications were searched initially based on the Boolean logic retrieval, but only 2,587 studies remained after duplicates were deleted. The titles and abstracts were screened, and 167 articles were selected for full-text assessment. Of these, 12 articles were excluded, and 155 trials were finally included in the present study based on the eligible criteria. The detailed literature search process is illustrated in Figure 1.

### Characteristics of the included studies

The present study comprised a sample size of 15,014 cases. The male participants accounted for 64% of all participants. Eight combined interventions were included: azithromycin injection alone (AZ), azithromycin with Xiyanping injection (AZ + XYP) (Yuan and Sun, 2011; Jiang, 2013; Wang H. Y., 2013; Luo, 2014; Ning, 2014; Yang, 2014; Deng and Li, 2015; Liang et al., 2015; Tang, 2015; Li and Shao, 2016; Shu and Liu, 2016; Wang, 2016; Wang et al., 2016b; Liu et al., 2017; Ruan et al., 2017; Qin, 2018; Wei J. H., 2018; Zhao et al., 2018; Wang, 2019; Wang L., 2020; Xu et al., 2020; Zhang Y. X. et al., 2021; Ji, 2022; Meng, 2022; Wang and Wang, 2022), azithromycin with Reduning injection (AZ + RDN) (Du and Wang, 2011; Liu, 2011; An et al., 2012; Peng, 2013; Chen and Ma, 2014; Gao, 2014; Wang et al., 2014; Gao X. Q., 2015; Shi et al., 2015; Zhou M., 2015; Xia and Pan, 2016; Hou, 2017; Huang, 2017; Tan D. D., 2017; Tao, 2017; Zhang Y., 2017; Li and Li, 2018; Zhang and Gao, 2018; Zhu and Luo, 2018; Li S. X., 2020; Xu, 2020; Zhou, 2020; Shu et al., 2021; Chen et al., 2022; Yu, 2022; Gao, 2023), azithromycin with Tanreqing injection (AZ + TRQ) (Chen, 2008; Shi, 2009; Yan et al., 2010; Chen and Liu, 2011; Li, 2011; Luo and Wang, 2011; Xiao, 2011; Xiong and Peng, 2011; Xu, 2011; Zhang, 2011; Zhang et al., 2011; Zhang, 2012; Bo, 2013; Jiang et al., 2013; Li, 2013; Liu, 2013; Wang et al., 2013; Wang and Wu, 2013; Wang P. H., 2013; Wu, 2013; Hu, 2014; Huang, 2014; Liang, 2014; Sheng, 2014; Zhen, 2014; Zhong et al., 2014; Cai, 2015; Deng, 2015; Gao L. Z., 2015; Gao P. J., 2015; Hu, 2015; Peng et al., 2015; Yi, 2015; Zhou W. Z., 2015; Chen et al., 2016; Chen, 2016; Jiang, 2016; Liang, 2016; Liu and Ye, 2016; Liu, 2016; Qi, 2016; Quan, 2016; Wang et al., 2016a; Zhang et al., 2016; Zhang, 2016; Gao, 2017; Lin et al., 2017; Liu, 2017; Lu X. H., 2017; Lu Z., 2017; Tan X., 2017; Xu, 2017; Zhang H. X., 2017; Wang and He, 2018; Wang Y. N., 2018; Wei C. X., 2018; Zhao, 2018; Liu, 2019; Gao, 2020; Li T., 2020; You, 2020; Ding and Yang, 2021; Gao, 2021; Jin, 2021; Liu, 2021; Yuan, 2021; Zhang, 2021; Zhang, 2022; Zhang, 2023), azithromycin with Yanhuning injection (AZ + YHN) (Chen et al., 2007; Hu, 2008; Xi, 2010; Cao, 2011; Chen, 2011; Liu and Tao, 2011; Song, 2011; Su, 2011; Li, 2012; Zhou, 2012; Lin, 2013; Jiang and Zhang, 2014; Wen, 2014; Zhang and Shi, 2014; Han, 2015; Ma, 2015; Chen, 2017; Jiang et al., 2017; Li, 2017; Liu and Gou, 2017; Zhu, 2017; Li, 2018; Li et al., 2018; Han, 2019), azithromycin with Xixinnao injection (AZ + XXN) (Yao, 2011; Gao, 2013; Song and Yang, 2014; Zhang, 2014; Meng and Wang, 2017; Zhang, 2020), azithromycin with Qingkailing injection (AZ + QKL) (Meng, 2014; Wang X. Q., 2018), and azithromycin with Chuanhuning injection (AZ + CHN) (Sun, 2005; Liu, 2008; An, 2009). All RCTs were conducted in China and published between 2004 and 2023. For the outcomes, 144 studies (93.0%) reported clinical effectiveness rate, 140 studies (90.3%) evaluated the disappearance time of cough, 128 studies (82.6%) reported disappearance time of pulmonary rale, 141 studies (91.0%) assessed disappearance time of fever, 76 studies (49.0%) assessed average hospitalization time, and 51 studies (33.0%) reported disappearance time of pulmonary shadows in *X*-ray. The details of the baseline characteristics of the studies are provided in Supplementary Material S6.

### Methodological quality

Of the included studies, 78 were considered to have a “low risk” of bias, 75 were rated as having “some concerns,” and 2 were classified as “high risk.”

Although all 155 studies adopted a randomized approach, two studies were found to be at “high risk” due to flawed randomization methods, and 76 studies that used correct randomization methods (computer-generated random numbers, reference to a random number table, coin tossing, throwing dice, or drawing lots) were rated as “low risk,” according to RoB 2.0

In most studies, allocation blinding was not reported: three RCTs mentioned the blinding of participants or personnel, but allocation blinding was unclear, and none of these studies were double-blinded (Lu Z., 2017; Li, 2018; Chen, 2017). Meanwhile, none of these 155 studies explicitly mentioned allocation concealment, so all the included studies were rated as “unclear risk.”

In terms of bias due to deviations from intended interventions, all included studies reported no deviations from allocated interventions and used an appropriate method to analyze treatment effects. Hence, all studies were regarded as “low risk.”

In terms of bias due to missing outcome data and bias in the measurement of the outcome, we could get complete data in all studies. Moreover, the measurement or determination of the outcomes in the two groups is consistent and objective; hence, all studies were evaluated as “low risk.”

**FIGURE 2:**
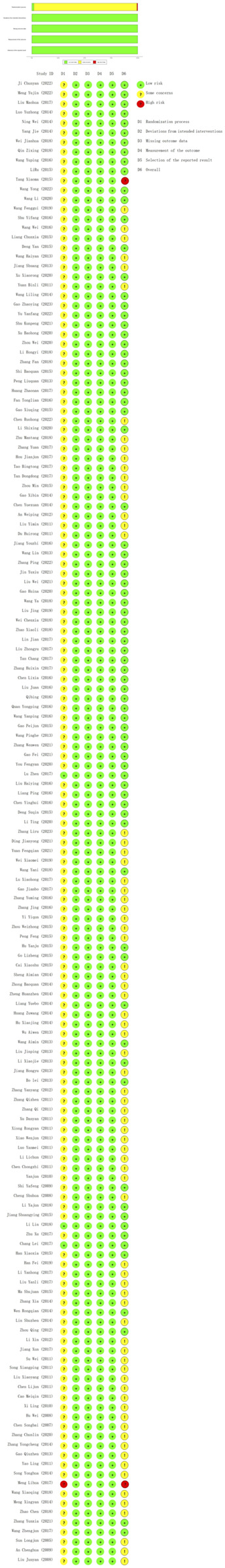
Risk of bias summary.

As for the bias in the selection of the reported results, all RCTs were rated as “low risk.” The results of the risk of bias for the included studies are shown in Figure 2.

### Network meta-analysis

**FIGURE 3:**
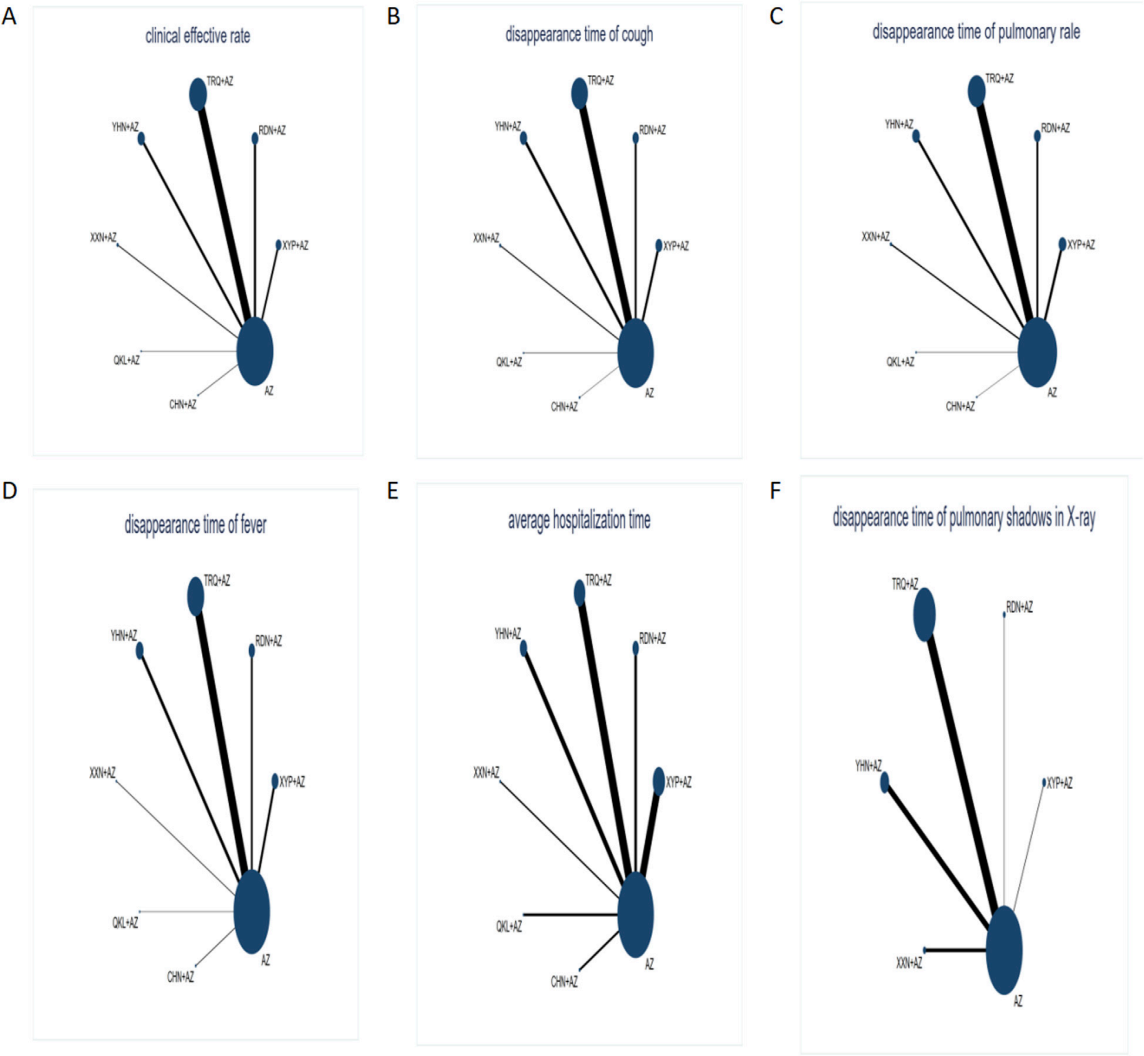
Network graph of different outcomes: **(A)** clinical effectiveness rate; **(B)** disappearance time of cough; **(C)** disappearance time of pulmonary rale; **(D)** disappearance time of fever; **(E)** average hospitalization time; and **(F)** disappearance time of pulmonary shadows in X-ray. The sizes of nodes and edges display the number of patients receiving the treatment and the number of studies for the comparison, respectively. Each intervention was shown by a circular node, and each connection represented a contrast. The diameter of the circular node was positively correlated with the number of patients included, and the line thickness was positively related to the number of direct comparisons.

Network graphs comparing AZ plus CMIs for MPP patients in each of the six outcomes are shown in Figure 3. The network graphs were generated using STATA 15.0. Each intervention was shown by a circular node, and each connection represented a contrast. The diameter of the circular node was positively correlated with the number of patients included, and line thickness was positively related to the number of direct comparisons.

Figure 3 shows that AZ was used as the comparator arm in all studies, but as there was no direct comparison between any two interventions, no closed loop existed. As a result, an inconsistency test was not required for this study; the consistency model was chosen to build Bayesian models. Based on the results presented in Table 2, the clinical effectiveness rate chose the fixed-effect model, and the remaining outcomes use the random-effect model.

**TABLE 2:** Comparison of DIC and *I* ^*2*^ between the random- and fixed-effect models.

Outcome	Model	Dbar	PD	DIC	I^2^(%)
Clinical effectiveness rate	Fixed-effect	211.96	153.01	364.97	0
	Random-effect	211.73	153.78	364.51	0
Disappearance time of cough	Fixed-effect	1720.03	148.54	1868.58	84
	Random-effect	278.06	262.50	540.57	0
Disappearance time of pulmonary rale	Fixed-effect	3177.38	138.22	3315.60	92
	Random-effect	250.44	237.91	488.35	0
Disappearance time of fever	Fixed-effect	3047.04	150.39	3197.44	91
	Random-effect	282.23	263.80	546.03	0.4
Average hospitalization time	Fixed-effect	361.36	45.24	406.59	79
	Random-effect	77.21	73.30	150.51	3
Disappearance time of pulmonary shadows in X-ray	Fixed-effect	387.05	57.31	444.36	74
	Random-effect	96.17	87.83	184.00	0

**FIGURE 4:**
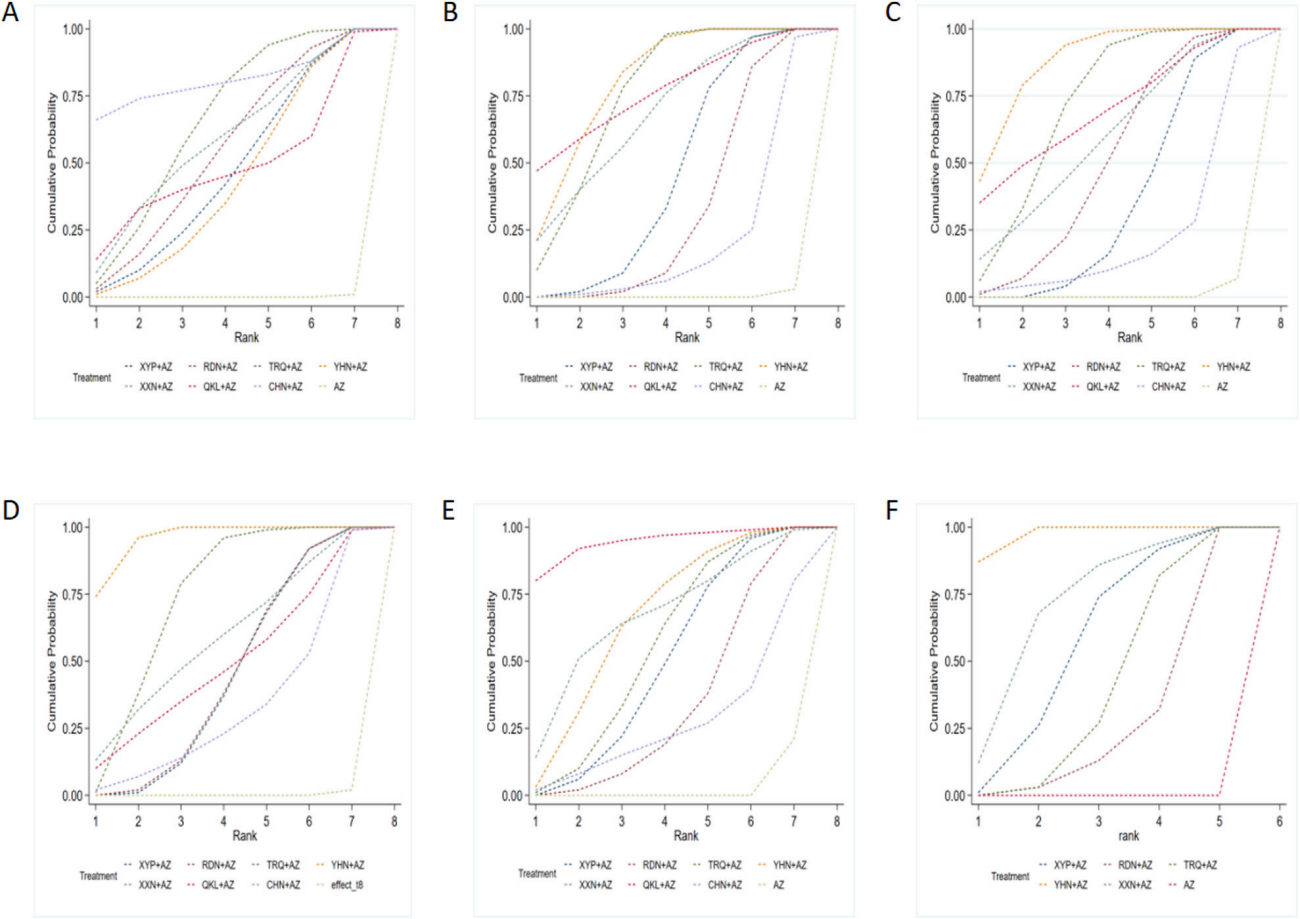
Surface under the cumulative ranking (SUCRA) probabilities of different interventions: **(A)** clinical effectiveness rate; **(B)** disappearance time of cough; **(C)** disappearance time of pulmonary rale; **(D)** disappearance time of fever; **(E)** average hospitalization time; and **(F)** disappearance time of pulmonary shadows in X-ray. AZ, azithromycin injection; XYP, Xiyanping injection; RDN, Reduning injection; TRQ, Tanreqing injection; YHN, Yanhuning injection; XXN, Xixinnao injection; QKL, Qingkailing injection; CHN, Chuanhuning injection.

The results of the ranking probabilities based on SUCRA are shown in Table 3 and Figure 4. ORs (95% CIs)/(MDs) of all interventions for the six outcomes in our BNMA are shown in Table 4.

**TABLE 3:** Ranking probability of interventions.

Intervention	Clinical effectiveness rate		Disappearance time of cough		Disappearance time of pulmonary rale		Disappearance time of fever		Average hospitalization time		Disappearance time of pulmonary shadows in *X*-ray	
	SUCRA (%)	Rank	SUCRA (%)	Rank	SUCRA (%)	Rank	SUCRA (%)	Rank	SUCRA (%)	Rank	SUCRA (%)	Rank
XYP + AZ	46.78	6	45.56	5	36.32	6	44.41	6	50.09	5	58.54	3
RDN + AZ	54.94	4	32.95	6	51.52	5	44.62	5	35.19	6	29.70	5
TRQ + AZ	65.77	2	75.06	3	71.97	2	73.50	2	55.95	4	42.35	4
YHN + AZ	43.71	7	80.01	1	87.77	1	95.70	1	66.53	3	97.34	1
XXN + AZ	59.05	3	68.48	4	59.57	4	58.81	3	66.96	2	72.05	2
QKL + AZ	48.68	5	76.73	2	69.28	3	49.61	4	94.60	1	-	-
CHN + AZ	80.88	1	20.8	7	22.57	7	33.06	7	27.61	7	-	-
AZ	0.18	8	0.41	8	1.01	8	0.30	8	3.07	8	0.30	6

**TABLE 4:** Outcomes of network meta-analysi**s**.

A: Network meta-analysis comparisons for clinical effectiveness rate							
XYP + AZ							
0.94 (0.59, 1.48)	RDN + AZ						
0.87 (0.59, 1.3)	0.93 (0.64, 1.35)	TRQ + AZ					
1.02 (0.65, 1.6)	1.09 (0.71, 1.68)	1.18 (0.82, 1.67)	YHN + AZ				
0.90 (0.44, 1.76)	0.96 (0.47, 1.85)	1.04 (0.53, 1.91)	0.88 (0.44,1.68)	XXN + AZ			
1.06 (0.2, 3.9)	1.14 (0.22, 4.12)	1.22 (0.24, 4.33)	1.04 (0.2, 3.8)	1.18 (0.22, 4.88)	QKL + AZ		
0.38 (0.01, 2.86)	0.41 (0.01, 3.06)	0.44 (0.02, 3.18)	0.37 (0.01, 2.81)	0.42 (0.01, 3.46)	0.36 (0.01, 4.74)	CHN + AZ	
**4.83 (3.47, 6.84)**	**5.17 (3.82, 7.13)**	**5.56 (4.56, 6.87)**	**4.74 (3.56, 6.39)**	**5.38 (3.03, 10.12)**	**4.58 (1.3, 22.71)**	**12.67 (1.76, 341.09)**	AZ

B: Network meta-analysis comparisons for the disappearance time of cough							
XYP + AZ							
−0.25 (−0.9, 0.4)	RDN + AZ						
0.47 (−0.04, 0.98)	**0.71 (0.17, 1.26)**	TRQ + AZ					
0.55 (−0.08, 1.18)	**0.79 (0.14, 1.44)**	0.08 (−0.44, 0.61)	YHN + AZ				
0.39 (−0.69, 1.48)	0.64 (−0.46, 1.74)	−0.07 (−1.1, 0.97)	−0.15 (−1.25, 0.92)	XXN + AZ			
0.68 (−0.96, 2.35)	0.92 (−0.72, 2.59)	0.21 (−1.4, 1.82)	0.13 (−1.51, 1.8)	0.29 (−1.59, 2.16)	QKL + AZ		
−0.81 (−2.08, 0.49)	−0.56 (−1.84, 0.76)	**−1.27 (-2.51, -0.03)**	**−1.35 (-2.63, -0.06)**	−1.21 (−2.75, 0.37)	−1.48 (−3.48, 0.47)	CHN + AZ	
**−1.99 (-2.43, -1.54)**	**−1.74 (-2.22, -1.26)**	**−2.45 (-2.73, -2.19)**	**−2.54 (-2.99, -2.09)**	**−2.38 (-3.37, -1.39)**	**−2.67 (-4.27, -1.08)**	−1.18 (−2.39, 0.02)	AZ

C: Network meta-analysis comparisons for the disappearance time of pulmonary rale							
XYP + AZ							
0.24 (−0.4, 0.88)	RDN + AZ						
**0.53 (0.02, 1.03)**	0.28 (−0.26, 0.83)	TRQ + AZ					
**0.79 (0.17, 1.39)**	0.55 (−0.1, 1.2)	0.26 (−0.26, 0.77)	YHN + AZ				
0.35 (−0.72, 1.44)	0.11 (−0.98, 1.21)	−0.18 (−1.2, 0.85)	−0.44 (−1.52, 0.66)	XXN + AZ			
0.58 (−0.93, 2.1)	0.33 (−1.22, 1.89)	0.05 (−1.46, 1.55)	−0.21 (−1.74, 1.3)	0.22 (−1.54, 1.98)	QKL + AZ		
−0.66 (−2.18, 0.86)	−0.91 (−2.41, 0.62)	−1.19 (−2.67, 0.29)	−1.45 (−2.96, 0.07)	−1.01 (−2.79, 0.72)	−1.24 (−3.32, 0.81)	CHN + AZ	
**−1.74 (-2.17, -1.32)**	**−1.99 (-2.47, -1.51)**	**−2.28 (-2.55, -2)**	**−2.53 (-2.96, -2.1)**	**−2.1 (-3.08, -1.11)**	**−2.33 (-3.79, -0.84)**	−1.09 (−2.53, 0.37)	AZ

D: Network meta-analysis comparisons for the disappearance time of fever							
XYP + AZ							
0 (−0.47, 0.47)	RDN + AZ						
0.33 (−0.05, 0.71)	0.33 (−0.07, 0.73)	TRQ + AZ					
**0.72 (0.27, 1.18)**	**0.72 (0.25, 1.19)**	**0.4 (0.02, 0.76)**	YHN + AZ				
0.17 (−0.85, 1.16)	0.17 (−0.85, 1.18)	−0.16 (−1.14, 0.8)	−0.55 (−1.57, 0.44)	XXN + AZ			
0.01 (−1.14, 1.16)	0.01 (−1.15, 1.15)	−0.32 (−1.45, 0.8)	−0.71 (−1.86, 0.43)	−0.16 (−1.61, 1.31)	QKL + AZ		
−0.28 (−1.28, 0.72)	−0.27 (−1.28, 0.74)	−0.6 (−1.57, 0.36)	**−0.99 (-1.99, 0)**	−0.44 (−1.78, 0.89)	−0.29 (−1.74, 1.15)	CHN + AZ	
**−1.36 (-1.68, -1.03)**	**−1.36 (-1.7, -1.01)**	**−1.68 (-1.88, -1.49)**	**−2.08 (-2.39, -1.75)**	**−1.52 (-2.46, -0.56)**	**−1.37 (-2.46, -0.25)**	**−1.08 (-2.02, -0.14)**	AZ

E: Network meta-analysis comparisons for average hospitalization time							
XYP + AZ							
−0.45 (−1.82, 0.89)	RDN + AZ						
0.14 (−1.01, 1.23)	0.59 (−0.77, 1.95)	TRQ + AZ					
0.5 (−1, 1.93)	0.95 (−0.7, 2.61)	0.35 (−1.12, 1.83)	YHN + AZ				
0.75 (−2.23, 3.65)	1.2 (−1.86, 4.24)	0.6 (−2.39, 3.57)	0.26 (−2.88, 3.37)	XXN + AZ			
2.84 (−0.36, 5.98)	3.29 (−0.02, 6.57)	2.7 (−0.52, 5.87)	2.34 (−0.95, 5.6)	2.09 (−2.07 , 6.29)	QKL + AZ		
−1.18 (−4.35, 2)	−0.74 (−3.96, 2.53)	−1.33 (−4.47, 1.85)	−1.69 (−4.97, 1.63)	−1.94 (−6.11, 2.23)	−4.03 (−8.33, 0.31)	CHN + AZ	
**−2.45 (-3.25, -1.68)**	**−2 (-3.1, -0.89)**	**−2.6 (-3.39, -1.8)**	**−2.95 (-4.2, -1.71)**	**−3.2 (-6.05, -0.33)**	**−5.29 (-8.35, -2.23)**	−1.26 (−4.34, 1.78)	AZ

F: Network meta-analysis comparisons for the disappearance time of pulmonary shadows in X-ray					
XYP + AZ					
−0.83 (−2.43, 0.74)	RDN + AZ				
−0.41 (−1.51, 0.73)	0.43 (−0.82, 1.7)	TRQ + AZ			
**1.56 (0.33, 2.84)**	**2.39 (1.03, 3.79)**	**1.97 (1.15, 2.81)**	YHN + AZ		
0.54 (−1.41, 2.51)	1.37 (−0.66, 3.37)	0.95 (−0.74, 2.68)	−1.01 (−2.79, 0.78)	XXN + AZ	
**−2.79 (-3.84, -1.75)**	**−1.96 (-3.14, -0.76)**	**−2.38 (-2.82, -1.97)**	**−4.35 (-5.08, -3.65)**	**−3.34 (-5, -1.7)**	AZ

Notes: AZ, azithromycin injection; XYP, Xiyanping injection; RDN, Reduning injection; TRQ, Tanreqing injection; YHN, Yanhuning injection; XXN, Xixinnao injection; QKL, Qingkailing injection; CHN, Chuanhuning injection. Significant effects are printed in bold.

## Outcome measures

### Clinical effectiveness rate

A total of 144 included RCTs reported the clinical effectiveness rate, referring to eight interventions. Table 4-A details the effectiveness of the comparison of different interventions by ORs and the corresponding 95% CIs in the BNMA. Compared to AZ alone, the combinations AZ + XYP, AZ + RDN, AZ + TRQ, AZ + YHN, AZ + XXN, AZ + QKL, and AZ + CHN demonstrated superior clinical efficacy.

The ranking results of interventions by SUCRA in Figure 4A and Table 4 showed that strategies of AZ + CHN (ranking to 1) may have relative advantages in the treatment of MPP.

### Disappearance time of cough

A total of 140 articles reported the disappearance time of cough and evaluated eight interventions. According to Table 4-B, compared with AZ alone, AZ + XYP, AZ + RDN, AZ + TRQ, AZ + YHN, AZ + XXN, and AZ + QKL had better efficacy to reduce disappearance time of cough. In addition, YHN + AZ was better than RDN + AZ and CHN + AZ; TRQ + AZ was better than RDN + AZ and CHN + AZ in reducing the disappearance time of cough.

Based on the ranking analysis (Figure 4B and Table 3), AZ + YHN attained the first rank, AZ + QKL was the second, and AZ alone was associated with the lowest probability of reducing the disappearance time of cough.

### Disappearance time of pulmonary rale

In terms of disappearance time of pulmonary rale, 7 treatments with 128 RCTs were compared with AZ. The network comparisons displayed in Table 4 C suggested that there were six interventions (AZ + XYP, AZ + RDN, AZ + TRQ, AZ + YHN, AZ + XXN, and AZ + QKL) that could improve the disappearance time of pulmonary rale. Moreover, AZ + TRQ and AZ + YHN were significantly better than XYP + AZ in reducing pulmonary rales.

According to the SUCRA probabilities (Figure 4C and Table 3), the strategies of AZ + YHN (ranking to 1) may have relative advantages in the disappearance time of pulmonary rale.

### Disappearance time of fever

A total of 141 studies had reported the disappearance time of fever, including eight interventions. Table 4 D reveals that AZ combined with XYP, RDN, TRQ, YHN, XXN, QKL, and CHN was significantly better than AZ alone in reducing the disappearance time of fever. AZ + YHN was significantly more effective than AZ + XYP, AZ + RDN, AZ + TRQ, and AZ + CHN in improving the disappearance time of fever.

According to the SUCRA results presented in Figure 4D and Table 3, the strategies of AZ + YHN (ranking to 1) may be the best option to improve the disappearance time of fever.

### Average hospitalization time

Seventy-six RCTs reported an average hospitalization time for azithromycin combined with seven CMIs in the treatment of MPP. The outcomes of the BNMA showed that compared to AZ, AZ + XYP, AZ + RDN, AZ + TRQ, AZ + YHN, and AZ + QKL had better efficacy in shortening average hospitalization time; the specific outcomes are shown in Table 4 E.

Treatment ranking based on SUCRA values, the strategies of AZ + QKL (ranking to 1), may have relative advantages in shortening average hospitalization time. Detailed information is shown in Figure 4E and Table 3.

### Disappearance time of pulmonary shadows in *X*-ray

Of the included studies, 78 were considered to have estimated in 51 RCTs. According to Table 4 F, AZ combined with five CMIs: AZ + XYP, AZ + RDN, AZ + TRQ, AZ + YHN, and AZ + XXN were more effective than AZ alone. Furthermore, YHN + AZ was significantly better than XYP + AZ, RDN + AZ, and TRQ + AZ in the disappearance time of pulmonary shadows in *X*-ray.

The ranking by SUCRA (Figure 4F and Table 3) showed that AZ + YHN (ranking to 1) may have relative advantages in shortening the disappearance time of pulmonary shadows in *X*-ray.

### Adverse reaction assessment

Due to the absence of unified criteria in different clinical trials, we listed the definite cases of adverse events in each trial. Among the 155 RCTs, 98 studies (63.23%) reported adverse reactions during treatment, involving 9,222 patients. The frequency was 484/4,659 (10.39%) in the experimental group and 731/4,563 (16.02%) in the control group. Out of the RCTs, 146 provided detailed descriptions, which were summarized into six types of adverse reaction events: digestive system issues, skin rash, dizziness/headache, pain at the injection site, liver dysfunction, and others. The incidence of different types of *adverse reactions* in different interventions is outlined in Supplementary Material S5, with digestive system issue reactions being the most prevalent among all competing interventions. All symptoms were alleviated after corresponding treatment and did not influence the RCTs.

### Publication bias

**FIGURE 5:**
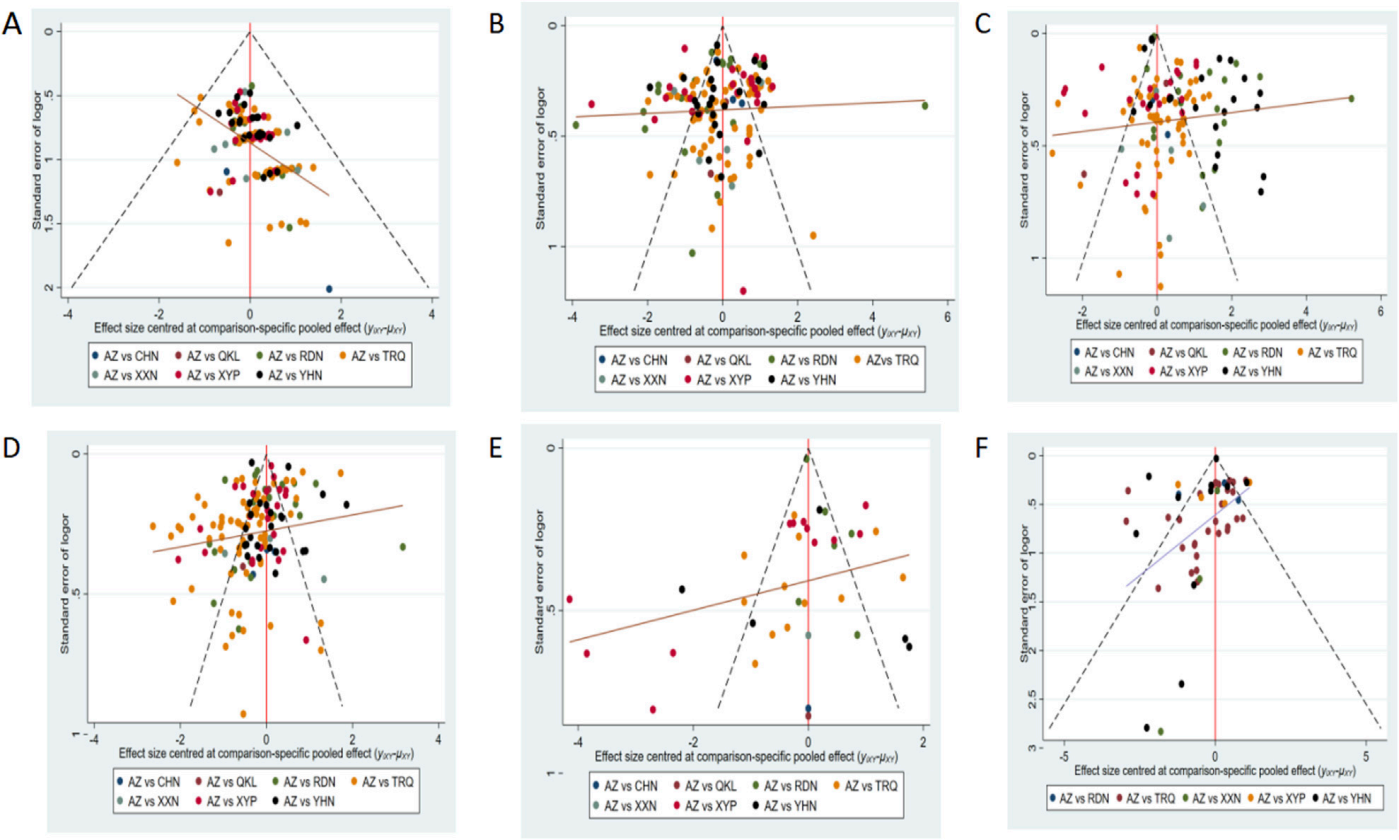
Funnel plot of outcomes: **(A)** clinical effectiveness rate; **(B)** disappearance time of cough; **(C)** disappearance time of pulmonary rale; **(D)** disappearance time of fever; **(E)** average hospitalization time; and **(F)** disappearance time of pulmonary shadows in X-ray. AZ, azithromycin injection; XYP, Xiyanping injection; RDN, Reduning injection; TRQ, Tanreqing injection; YHN, Yanhuning injection; XXN, Xixinnao injection; QKL, Qingkailing injection; CHN, Chuanhuning injection.

Comparison-adjusted funnel plots for different outcomes are displayed in Figure 5. Four funnel plots—showing the clinical effectiveness rate, disappearance time of cough, disappearance time of pulmonary rale, and disappearance time of fever—were generally visually symmetrical, indicating no publication bias. For the remaining outcomes, the funnel plots were not visually symmetrical, which revealed the presence of small sample size and publication bias.

## Discussion

Chinese medicine injections are formulated by extracting active metabolites from traditional Chinese botanical drugs using modern technology, which is known for its rapid action and high bioavailability, exhibiting a pharmacological effect characterized by “multi-metabolites, multi-targets” (Zhao et al., 2022). This innovative formulation addresses the traditional slow onset linked with Chinese botanical drugs by bypassing the need for oral administration, rendering them an efficient and dependable option for patients. In China, CMIs are widely utilized in clinical practice as a complementary treatment. However, the lack of direct comparisons between different types of CMIs often complicates the decision-making process for clinical physicians in selecting optimal therapy for patients with MPP. Traditional pairwise meta-analysis is limited to direct comparisons between two interventions and cannot comprehensively evaluate the efficacy across different treatments. Consequently, our study employs the BNMA to systematically evaluate the efficacy of CMIs for treating MPP in children. This BNMA was conducted to elucidate the best available evidence regarding the comparative effectiveness of various CMIs, aiming to provide guidance for physicians in clinical practice.

The present systematic review and network meta-analysis included 155 studies. Of these, 78 studies were categorized as “low risk” of bias, 75 studies had “some concerns,” and 2 studies were deemed “high risk.” The results of the network meta-analysis indicated that AZ + CHN ranked highest in improving the clinical effectiveness rate. AZ + YHN was found to be the most effective in alleviating symptoms in children with MPP. AZ + QKL showed the greatest reduction in average hospitalization time. Furthermore, compared to AZ alone, CMI therapies did not result in an increase in adverse reactions.

Chuanhuning, Yanhuning, and Xiyanping injections are derived from andrographolide extracted from the traditional Chinese botanical drug *Andrographis paniculata*. This botanical drug has a long history of use in treating respiratory tract infections and is recognized for its anti-inflammatory, anti-cancer, anti-obesity, anti-diabetic, and other medicinal properties (Hossain et al., 2021; Jiang et al., 2021). Chuanhuning injection (CHN) is among the initial batch of commercial Chinese polyherbal preparations designated for emergency use in national Chinese medicine hospitals, authorized by the China State Food and Drug Administration. It is predominantly employed in clinical settings for treating acute respiratory infections (Wu et al., 2015). An animal study has suggested that CHN enhances neointima formation by regulating the proliferation of smooth muscle cells, thereby promoting vascular intimal remodeling to suppress inflammatory responses (Guo et al., 2024). *In vitro* inhibition experiments have demonstrated that CHN exhibits inhibitory effects on 11 types of bacteria, including *Streptococcus*, Pneumococcus, and *Klebsiella pneumoniae*; it could enhance peripheral blood neutrophil and macrophage phagocytosis, increase serum lysozyme levels, and reduce endotoxin-induced fever (Xiaomeng et al., 2015). It is noteworthy that the China Drug and Food Administration reported nephrotoxicity risks associated with Chuanhuning injection (Feng et al., 2018). Consequently, CHN injection is less commonly used in pediatrics. Among the included RCTs, no nephrotoxicity reactions associated with Chuanhuning injection were reported.

Clinical studies have found that Yanhuning injection could significantly reduce the levels of IL-4, IL-10, IL-6, TNF-*α*, and IFN-*γ* in pediatric children, and Yanhuning injection influences both anti-inflammatory and pro-inflammatory cytokines, as well as the immunological function of children with MMP, showing a marked improvement in their inflammatory and immune states (Shang et al., 2022). Based on this study, azithromycin combined with Yanhuning injection shows promise as an effective treatment for improving symptoms in children with *Mycoplasma pneumoniae* pneumonia. Compared with other CMIs, Tanreqing injection primarily functions to decrease plasma levels of IL-8 and NE, thereby improving the response to airway inflammation and reducing mucus hypersecretion. It further improves the patients’ breath so that the clinical symptoms of cough can disappear in a short time (Li et al., 2010).

Evidence from the study indicates that the above seven types of CMIs are effective supplementary therapy for MPP. However, strict import and export controls in various countries present significant barriers to the global promotion of CMIs due to constraints in production technology and the complex composition of these products. Moreover, the mechanism of CMIs in the treatment of MPP is unclear and requires further investigation. More high-quality RCTs with strict design RCTs and larger sample sizes are needed to further corroborate the evidence.

## Limitation

There were limitations and shortcomings in our research. First, all RCTs were carried out in China, and the data from clinical studies in other languages were lacking, which may have caused the risk of bias. In addition, the quality of the RCTs included in this research was general, largely because merely three RCTs mentioned blinding. The poor quality of the methodology might contribute to an exaggerated curative effect and decreased reliability of the evidence. Moreover, only three articles focused on CHN and two articles on QKL were included. The small sample size also made it difficult to detect significant differences between the treatment and control groups.

## Conclusion

This study determined the efficacy of azithromycin combined with seven types of Chinese medicine injections. CHN may be the best adjunctive Chinese medicine injection for *Mycoplasma* pneumoniae pneumonia in children. Due to the potential risk of bias and limited RCTs, the results need to be treated with caution.

## Data Availability

The original contributions presented in the study are included in the article/Supplementary Material; further inquiries can be directed to the corresponding authors.

## References

[B1] An C. H. (2009). Observation of therapeutic efficacy in 44 cases of pediatric Mycoplasma pneumonia treated with azithromycin and Chuanhuning. Shandong Med. 49 (47), 52–53. (in Chinese). 10.3969/j.issn.1002-266X.2009.47.022

[B2] An W. P. Li R. Guo H. M. Xu B. G. (2012). Clinical study of azithromycin combined with Reduning for the treatment of Mycoplasma pneumonia in children. China Pract. Med. 7 (16), 28–29. (in Chinese). 10.3969/j.issn.1673-7555.2012.16.013

[B3] Beeton M. L. Zhang X. S. Uldum S. A. Bébéar C. Dumke R. Gullsby K. (2020). Mycoplasma pneumoniae infections, 11 countries in Europe and Israel, 2011 to 2016. Euro Surveill. 25 (2), 1900112. 10.2807/1560-7917.Es.2020.25.2.1900112 31964459 PMC6976882

[B4] Bo L. (2013). Efficacy of Tanreqing injection combined with azithromycin in the treatment of 60 children with Mycoplasma pneumonia. Chin. Pediatr. Integr. Traditional West. Med. 5 (05), 421–422. (in Chinese). 10.3969/j.issn.1674-3865.2013.05.017

[B5] Cai X. S. (2015). The effectiveness of treating Mycoplasma pneumonia. Clin. J. Chin. Med. 7 (19), 110–111. (in Chinese). 10.3969/j.issn.1674-7860.2015.19.063

[B6] Cao M. Q. (2011). Efficacy of azithromycin combined with Yanhuning in pediatric Mycoplasma pneumonia. Pract. J. Cardiac Cereb. Pneumal Vasc. Dis. 19 (04), 609–610. (in Chinese). 10.3969/j.issn.1008-5971.2011.04.057

[B7] Chen H. Z. Liu Y. (2011). Efficacy of azithromycin combined with Tanreqing to treat Mycoplasma pneumoniae pneumonia. China Health Ind. 8 (Z5), 40. (in Chinese). 10.16659/j.cnki.1672-5654.2011.z5.087

[B8] Chen L. (2017). Clinical observation of azithromycin combined with Yanhuning in the treatment of Mycoplasmal pneumonia in children. J. Yanan Univ. Sci. 15 (03), 45–47. (in Chinese). 10.3969/j.issn.1672-2639.2017.03.015

[B9] Chen L. J. (2011). Clinical effect of Yanhuning in 125 cases of Mycoplasma pneumonia. Guide China Med. 9 (08), 224–225. (in Chinese). 10.3969/j.issn.1671-8194.2011.08.173

[B10] Chen L. X. Tan Y. F. Zhang X. Z. (2016). Efficacy observation of Tanreqing combined with azithromycin for treating Mycoplasma pneumonia. China Mod. Med. 23 (14), 121–123. (in Chinese).

[B11] Chen R. H. Feng Y. C. Yan L. L. (2022). Effect of Reduning plus azithromycin sequential therapy in children with Mycoplasma pneumonia. Chin. J. Lung Dis. Ed. 15 (01), 88–90. (in Chinese). 10.3877/cma.j.issn.1674-6902.2022.01.025

[B12] Chen S. B. Yang J. Song Y. (2007). Yanhuning combined with azithromycin for pediatric Mycoplasma pneumonia. J. Aerosp. Med. (01), 21–22. (in Chinese). 10.3969/j.issn.2095-1434.2007.01.010

[B13] Chen S. H. (2008). Clinical observation of Tanreqing injection in pediatric treatment of Mycoplasma pneumonia. Chin. J. Pract. Med. 35 (16), 82–83. (in Chinese). 10.3760/cma.j.issn.1674-4756.2008.16.061

[B14] Chen Y. H. (2016). Clinical efficacy and safety study of Tanreqing combined with azithromycin sequential therapy in the treatment of Mycoplasma pneumonia. Mod. J. Integr. Traditional Chin. West. Med. 25 (14), 1570–1571+1585. (in Chinese). 10.3969/j.issn.1008-8849.2016.14.033

[B15] Chen Y. X. Ma H. M. (2014). Effect of Reduning injection combined with sequential therapy using azithromycin on inflammatory cytokines and immune function in pediatric Mycoplasma pneumonia. J. Hainan Med. Univ. 20 (12), 1702–1704. (in Chinese). 10.13210/j.cnki.jhmu.20141010.021

[B16] China N. H. C. o.t.P. s.R. o. (2023). Guidelines for the diagnosis and treatment of Mycoplasma pneumoniae pneumonia in children(2023 edition). Int. J. Epidemiol. Infect. Dis. (02), 79–85.

[B17] Chun H. S. Choi S. H. Song H. S. (2021). A meta-analysis of treatment effects on viral pneumonia using TCM injections specified in the clinical guideline for COVID-19 in China. J. Pharmacopuncture 24 (3), 107–121. 10.3831/kpi.2021.24.3.107 34631193 PMC8481675

[B18] Deng S. Q. (2015). Impact of Tanreqing injection and azithromycin on C-reactive protein and myocardial enzyme levels in pediatric Mycoplasma pneumonia. Mod. J. Integr. Traditional Chin. West. Med. 24 (20), 2226–2228. (in Chinese). 10.3969/j.issn.1008-8849.2015.20.022

[B19] Deng Y. Li L. (2015). Efficacy of Xiyanping injection and azithromycin for sequential treatment in children with Mycoplasma pneumonia. Hebei Med. 21 (10), 1613–1616. (in Chinese). 10.3969/j.issn.1006-6233.2015.10.011

[B20] Ding J. Y. Yang L. L. (2021). Clinical effect of Tanreqing injection combined with azithromycin in sequential treatment of pediatric Mycoplasma pneumoniae pneumonia. Fertil. Health 19, 3–4. (in Chinese).

[B21] Du H. R. Wang Y. M. (2011). Clinical observation of azithromycin sequential therapy combined with Reduning injection for pediatric Mycoplasma pneumonia. Hebei Med. J. 33 (19), 2944–2945. (in Chinese). 10.3969/j.issn.1002-7386.2011.19.032

[B22] Feng X. Fang S. N. Gao Y. X. Liu J. P. Chen W. (2018). Current research situation of nephrotoxicity of Chinese herbal medicine. Zhongguo Zhong Yao Za Zhi 43 (3), 417–424. 10.19540/j.cnki.cjcmm.2018.0009 29600603

[B23] Gao F. (2021). Study on sequential azithromycin combined with tanreqing treating mycoplasma pneumoniae pneumonia in children to improve clinical therapeutic effect. Syst. Med. 6 (11), 135–137. (in Chinese). 10.19368/j.cnki.2096-1782.2021.11.135

[B24] Gao H. N. (2020). Effect of Tanreqing injection and azithromycin in treating pediatric Mycoplasma pneumonia. Med. J. Chin. People's Health 32 (18), 74–76. (in Chinese). 10.3969/j.issn.1672-0369.2020.18.030

[B25] Gao J. B. (2017). The curative effect of Tanreqing combined with azithromycin on lobar pneumonia caused by pediatric Mycoplasma infection. Shaanxi J. Traditional Chin. Med. 38 (04), 452–453. (in Chinese). 10.3969/j.issn.1000-7369.2017.04.021

[B26] Gao L. Z. (2015a). Clinical observation of azithromycin combined with Tanreqing for pediatric Mycoplasma pneumonia. Chin. J. Mod. Drug Appl. 9 (03), 86–87. (in Chinese). 10.14164/j.cnki.cn11-5581/r.2015.03.063

[B27] Gao P. J. (2015b). Efficacy of azithromycin combined with Tanreqing injection in 50 children patients with Mycoplasma pneumonia. Chin. J. Mod. Drug Appl. (9), 142–143. (in Chinese). 10.14164/j.cnki.cn11-5581/r.2015.09.100

[B28] Gao Q. Z. (2013). Combination of azithromycin and Xixinnao injection in treating 64 cases of Mycoplasma pneumonia in children. Chinese-foreign Women's Health (5), 59. (in Chinese).

[B29] Gao X. B. (2014). Efficacy of azithromycin combined with Reduning injection in acute tracheobronchitis caused by mycoplasma pneumoniae infection. China Pract. Med. 9 (14), 140–142. (in Chinese).

[B30] Gao X. Q. (2015c). Combination of reduning and azithromycin in pneumonia treatment. J. Aerosp. Med. 26 (02), 219+224. (in Chinese).

[B31] Gao Z. Y. (2023). Clinical effect of the Reduning injection combined with azithromycin in treating pediatric Mycoplasma pneumonia and its effect on the level of inflammatory factors. Chin. J. Clin. Ration. Drug Use 16 (1), 114–117. (in Chinese). 10.15887/j.cnki.13-1389/r.2023.01.035

[B32] Guo Q. Li J. Wang Z. Wu X. Jin Z. Zhu S. (2024). Potassium dehydroandrographolide succinate regulates the MyD88/CDH13 signaling pathway to enhance vascular injury-induced pathological vascular remodeling. Chin. J. Nat. Med. 22 (1), 62–74. 10.1016/s1875-5364(24)60562-5 38278560

[B33] Han F. (2019). Efficacy analysis of Yanhuning combined with azithromycin for treating Mycoplasma pneumonia. Diabetes world 16 (11), 60. (in Chinese).

[B34] Han X. X. (2015). The clinical efficacy of Yanhunin injection for the treatment of pediatric Mycoplasma pneumonia was analyzed. World Latest Med. Inf. 15 (60), 51+64. (in Chinese). 10.3969/j.issn.1671-3141.2015.60.044

[B35] Heinrich M. Jalil B. Abdel-Tawab M. Echeverria J. Kulić Ž. McGaw L. J. (2022). Best Practice in the chemical characterisation of extracts used in pharmacological and toxicological research-The ConPhyMP-Guidelines. Front. Pharmacol. 13, 953205. 10.3389/fphar.2022.953205 36176427 PMC9514875

[B36] Hong K. B. Choi E. H. Lee H. J. Lee S. Y. Cho E. Y. Choi J. H. (2013). Macrolide resistance of Mycoplasma pneumoniae, South Korea, 2000-2011. Emerg. Infect. Dis. 19 (8), 1281–1284. 10.3201/eid1908.121455 23876792 PMC3739509

[B37] Hossain S. Urbi Z. Karuniawati H. Mohiuddin R. B. Moh Qrimida A. Allzrag A. M. M. (2021). Andrographis paniculata (burm. F.) wall. Ex nees: an updated review of phytochemistry, antimicrobial pharmacology, and clinical safety and efficacy. Life (Basel) 11 (4), 348. 10.3390/life11040348 33923529 PMC8072717

[B38] Hou J. J. (2017). Evaluation of efficacy and safety of Reduning injection combined with azithromycin sequential therapy in children with Mycoplasma pneumoniae. J. Aerosp. Med. 28 (06), 726–728. (in Chinese). 10.3969/j.issn.2095-1434.2017.06.042

[B39] Hu W. (2008). Yanhuning adjuvant treatment of pediatric Mycoplasma pneumonia in 50 cases. China Pharm. (05), 50. (in Chinese). 10.3969/j.issn.1006-4931.2008.05.035

[B40] Hu X. J. (2014). Efficacy on mycoplasma pneumonia treated with azithromycin and tanreqing injection in children. World J. Integr. Traditional West. Med. 9 (05), 514–516. (in Chinese). 10.13935/j.cnki.sjzx.2014.05.036

[B41] Hu Y. J. (2015). 70 cases of infantile pneumonia mycoplasma pneumonia treatment effect. China Health Stand. Manag. 6 (01), 70–71. (in Chinese). 10.3969/j.issn.1674-9316.2015.01.057

[B42] Huang Z. N. (2017). Azithromycin combined with Reduning for treatment of Mycoplasma pneumoniae in pediatric pneumoniae. Clin. Med. 37 (07), 91–93. (in Chinese). 10.19528/j.issn.1003-3548.2017.07.042

[B43] Huang Z. W. (2014). Clinical observation of intravenous azithromycin combined with Tanreqing injection in treatment of children with mycoplasma pneumoniae pneumonia. Chin. J. New Clin. Med. 7 (10), 953–956. (in Chinese). 10.3969/j.issn.1674-3806.2014.10.20

[B44] Hutton B. Salanti G. Caldwell D. M. Chaimani A. Schmid C. H. Cameron C. (2015). The PRISMA extension statement for reporting of systematic reviews incorporating network meta-analyses of health care interventions: checklist and explanations. Ann. Intern Med. 162 (11), 777–784. 10.7326/m14-2385 26030634

[B45] Ji C. Y. (2022). Clinical efficacy study of Xiyanping injection combined with azithromycin in pediatric mycoplasma pneumoniae pneumonia. Med. Health (3), 0113–0116. (in Chinese).

[B46] Jiang H. Y. Qiu G. X. Shu X. P. (2013). Randomized controlled study of Tanreqing combined with azithromycin in pediatric acute Mycoplasma pneumonia. Liaoning J. Traditional Chin. Med. 40 (11), 2267–2268. (in Chinese).

[B47] Jiang M. Sheng F. Zhang Z. Ma X. Gao T. Fu C. (2021). Andrographis paniculata (Burm.f.) Nees and its major constituent andrographolide as potential antiviral agents. J. Ethnopharmacol. 272, 113954. 10.1016/j.jep.2021.113954 33610706

[B48] Jiang S. (2013). Azithromycin combined with Xiyanping injection treated 44 cases of pediatric mycoplasma pneumonia. China J. Pharm. Econ. (S1), 119–120. (in Chinese).

[B49] Jiang S. Y. Zhang C. X. (2014). The effect of azithromycin combined with Yanhunin treatment on Mycoplasma pneumonia in 45 cases. Chin. J. Prim. Med. Pharm. 21 (13), 2043–2044. (in Chinese). 10.3760/cma.j.issn.1008-6706.2014.13.055

[B50] Jiang X. Su Y. Liu X. Z. (2017). Yanhunin combined with azithromycin in 60 children with Mycoplasma pneumonia. alI Health 11 (4), 150–151. (in Chinese). 10.3969/j.issn.1009-6019.2017.04.211

[B51] Jiang Y. Z. (2016). Efficacy and safety of Tanreqing combined with azithromycin in pediatric Mycoplasma pneumonia. J. Traditional Chin. Med. Univ. Hunan 36 (A02), 1196–1197. (in Chinese).

[B52] Jin Y. X. (2021). Clinical effect of azithromycin combined with Tanreqing on children with Mycoplasma pneumoniae pneumonia. Chin. J. Mod. Drug Appl. 15 (18), 121–123. (in Chinese). 10.14164/j.cnki.cn11-5581/r.2021.18.045

[B53] Kawai Y. Miyashita N. Kubo M. Akaike H. Kato A. Nishizawa Y. (2013). Nationwide surveillance of macrolide-resistant Mycoplasma pneumoniae infection in pediatric patients. Antimicrob. Agents Chemother. 57 (8), 4046–4049. 10.1128/aac.00663-13 23716043 PMC3719750

[B54] Kim K. Jung S. Kim M. Park S. Yang H. J. Lee E. (2022). Global trends in the proportion of macrolide-resistant mycoplasma pneumoniae infections: a systematic review and meta-analysis. JAMA Netw. Open 5 (7), e2220949. 10.1001/jamanetworkopen.2022.20949 35816304 PMC9274321

[B55] Kutty P. K. Jain S. Taylor T. H. Bramley A. M. Diaz M. H. Ampofo K. (2019). Mycoplasma pneumoniae among children hospitalized with community-acquired pneumonia. Clin. Infect. Dis. 68 (1), 5–12. 10.1093/cid/ciy419 29788037 PMC6552676

[B56] Lee H. Yun K. W. Lee H. J. Choi E. H. (2018). Antimicrobial therapy of macrolide-resistant Mycoplasma pneumoniae pneumonia in children. Expert Rev. Anti Infect. Ther. 16 (1), 23–34. 10.1080/14787210.2018.1414599 29212389

[B57] Li C. L. (2011). Efficacy study of Tanreqing combined with azithromycin in pediatric Mycoplasma pneumonia. Guide China Med. 9 (31), 385. (in Chinese). 10.3969/j.issn.1671-8194.2011.31.304

[B58] Li H. Y. Li H. Y. (2018). Observation of the therapeutic efficacy of Reduning combined with azithromycin for pediatric Mycoplasma pneumonia. Henan Med. Res. 27 (7), 1252–1253. (in Chinese). 10.3969/j.issn.1004-437X.2018.07.050

[B59] Li L. (2018). Clinical effect of Yanhuning combined with azithromycin in pediatric Mycoplasma pneumonia. World Latest Med. Inf. 18 (07), 104. (in Chinese). 10.19613/j.cnki.1671-3141.2018.7.082

[B60] Li R. Shao L. J. (2016). Efficacy of Xiyanping injection and azithromycin in treating Mycoplasma pneumonia infection. road health 15 (05), 92. (in Chinese).

[B61] Li S. X. (2020a). Efficacy of Reduning and azithromycin in children with Mycoplasma pneumoniae pneumonia. Med. Forum 24 (14), 1989–1991. (in Chinese). 10.19435/j.1672-1721.2020.14.041

[B62] Li T. (2020b). Effect of Tanreqing injection and azithromycin on pediatric Mycoplasma pneumonia and its impact on C-reactive protein and myocardial enzyme levels. Cardiovasc. Dis. J. Integr. Traditional Chin. West. Med. 8 (27), 42–43. (in Chinese). 10.16282/j.cnki.cn11-9336/r.2020.27.030

[B63] Li W. Mao B. Wang G. Wang L. Chang J. Zhang Y. (2010). Effect of Tanreqing Injection on treatment of acute exacerbation of chronic obstructive pulmonary disease with Chinese medicine syndrome of retention of phlegm and heat in Fei. Chin. J. Integr. Med. 16 (2), 131–137. 10.1007/s11655-010-0131-y 20473738

[B64] Li X. (2012). Study of Azithromycin combined with Yanhuning in treatment of children with Mycoplasma pneumonia curative effect. China Health Vis. 20 (9), 374–375. (in Chinese).

[B65] Li X. J. (2013). Efficacy of azithromycin combined with Tanreqing in pediatric Mycoplasma pneumonia. Mod. Diagnosis and Treat. 24 (06), 1385. (in Chinese). 10.3969/j.issn.1001-8174.2013.06.133

[B66] Li Y. H. (2017). Clinical efficacy of azithromycin and Yanhuning in pediatric Mycoplasma pneumonia. World Latest Med. Inf. 17 (49), 107+111. (in Chinese). 10.3969/j.issn.1671-3141.2017.49.080

[B67] Li Y. J. Jia S. R. Zhang Y. L. (2018). The effect of Yanhuning combined with azithromycin in pediatric Mycoplasma pneumonia was observed. Chin. Baby (10), 119. (in Chinese). 10.3969/j.issn.1671-2242.2018.10.106

[B68] Liang C. X. Jiang X. F. De H. (2015). Observation of curative effect of azithromycin combined with xiyanping injection in the treatment of mycoplasma pneumonia in children. China J. Pharm. Econ. 10 (01), 46–48. (in Chinese).

[B69] Liang P. (2016). Analysis of the clinical effect of azithromycin combined with Tanreqing in children with Mycoplasma pneumoniae pneumonia. Chin. Foreign Med. Res. 14 (22), 27–28. (in Chinese). 10.14033/j.cnki.cfmr.2016.22.012

[B70] Liang Y. B. (2014). Clinical efficacy analysis of Tanreqing combined with azithromycin in 64 cases of pediatric Mycoplasma pneumonia. Mod. Diagnosis and Treat. 25 (08), 1738–1739. (in Chinese).

[B71] Lin J. Xu Z. Y. Xing D. W. (2017). Efficacy of tanreqing injection combined with azithromycin in treatment of mycoplasma pneumonia in children and its effects on inflammatory factors. Chin. Archives Traditional Chin. Med. 35 (9), 2418–2420. (in Chinese). 10.13193/j.issn.1673-7717.2017.09.061

[B72] Lin S. Z. (2013). The effect of azithromycin combined with Yanhuning in 156 cases of pediatric Mycoplasma pneumonia. Strait Pharm. J. 25 (9), 122–123. (in Chinese). 10.3969/j.issn.1006-3765.2013.09.055

[B73] Liu H. Y. Ye J. Y. (2016). Efficacy of Tanreqing injection and azithromycin in treating children with Mycoplasma pneumoniae pneumonia. J. New Chin. Med. 48 (01), 156–157. (in Chinese). 10.13457/j.cnki.jncm.2016.01.070

[B74] Liu J. (2016). Clinical observation of azithromycin combined with Tanreqing in pediatric Mycoplasma pneumoniae. Yiyao Qianyan 6 (10), 167–168. (in Chinese).

[B75] Liu J. (2019). Effect of Tanreqing combined with azithromycin sequential therapy in treating pediatric Mycoplasma pneumoniae pneumonia. Chin. Baby 20, 81–85. (in Chinese).

[B76] Liu J. P. (2013). Efficacy of azithromycin and Tanreqing combination in the treatment of Mycoplasma pneumonia in children. China Mod. Dr. 51 (32), 80–82. (in Chinese).

[B77] Liu J. Y. (2008). Efficacy observation of combined therapy with intravenous azithromycin and Chuanhuning in children with Mycoplasma pneumonia. China Pharm. Her. 5 (1), 67. (in Chinese).

[B78] Liu M. H. Liu C. J. Yu F. F. Li X. L. (2017). Effectiveness of Xiyanping combined with azithromycin in treating severe Mycoplasma pneumonia in children. ournal Pediatr. Pharm. 23 (12), 26–29. (in Chinese). 10.13407/j.cnki.jpp.1672-108X.2017.12.009

[B79] Liu W. (2021). Clinical effect of Tanreqing injection combined with azithromycin in sequential treatment of pediatric Mycoplasma pneumoniae pneumonia. Chin. J. Clin. Ration. Drug Use 14 (8), 122–123. (in Chinese). 10.15887/j.cnki.13-1389/r.2021.08.052

[B80] Liu X. Y. Tao Y. (2011). The effect of azithromycin combined with Yanhuning 48 cases of Mycoplasma pneumonia. China Mod. Dr. 49 (20), 71–72. (in Chinese). 10.3969/j.issn.1673-9701.2011.20.030

[B81] Liu Y. L. Gou C. F. (2017). The combination of azithromycin and Yanhuning was used for the treatment of Mycoplasma pneumonia in children. Psychol. Dr. 23 (12), 81–82. (in Chinese).

[B82] Liu Y. M. (2011). Clinical observation of azithromycin and Reduning in 88 children with Mycoplasma pneumonia. China Health Ind. 8 (20), 81. (in Chinese).

[B83] Liu Z. Y. (2017). Clinical study on Tanreqing Injection combined with azithromycin in sequential treatment of children with Mycoplasma pneumonia. Drugs and Clin. 32 (2), 237–240. (in Chinese). 10.7501/j.issn.1674-5515.2017.02.018

[B84] Lu X. H. (2017a). Comparison of the clinical efficacy of Tanreqing combined with azithromycin sequential therapy and azithromycin alone in pediatric Mycoplasma pneumoniae pneumonia. Chin. J. Mod. Drug Appl. 11 (07), 157–159. (in Chinese). 10.14164/j.cnki.cn11-5581/r.2017.07.079

[B85] Lu Z. (2017b). Analysis of 60 cases with Mycoplasma pneumoniae. Chin. Foreign Med. Res. 15 (14), 137–138. (in Chinese). 10.14033/j.cnki.cfmr.2017.14.077

[B86] Luo Y. M. Wang Y. (2011). Analysis of the effect of Tanreqing combined with azithromycin sequential therapy in pediatric Mycoplasma pneumoniae pneumonia. Guide China Med. 9 (02), 129–131. (in Chinese). 10.3969/j.issn.1671-8194.2011.02.094

[B87] Luo Y. Z. (2014). The curative effect discussion of azithromycin combined with xiyanping injection in the treatment of children with mycoplasma pneumonia. Med. Innovation China 16, 134–136. (in Chinese). 10.3969/j.issn.1674-4985.2014.16.044

[B88] Ma S. J. (2015). Evaluation of the efficacy of yanhuning and azithromycin in pediatric Mycoplasma pneumonia. J. Contemp. Clin. Med. 28 (01), 1210. (in Chinese).

[B89] Meng L. H. Wang H. H. (2017). Clinical analysis of Xixinnao combined with azithromycin for Mycoplasma pneumonia. Nei Mongol J. Traditional Chin. Med. 33 (05), 113. (in Chinese). 10.3969/j.issn.1006-0979.2014.05.126

[B90] Meng X. Y. (2014). Clinical observation of azithromycin combined with Qingkailing injection in 28 cases of pediatric Mycoplasma pneumonia. J. Qiannan Med. Coll. Natl. 27 (01), 18–20. (in Chinese).

[B91] Meng Y. J. (2022). Effect of xiyanping combined with azithromycin on mycoplasma pneumonia in children. Guide China Med. 20 (14), 53–56. (in Chinese).

[B92] Narita M. (2016). Classification of extrapulmonary manifestations due to mycoplasma pneumoniae infection on the basis of possible pathogenesis. Front. Microbiol. 7, 23. 10.3389/fmicb.2016.00023 26858701 PMC4729911

[B93] Ning W. (2014). The effect of xi yanping and azithromycin in pediatric mycoplasma pneumonia. Chin. Foreign Med. Res. (35), 146. (in Chinese).

[B94] Niu L. Xiao L. Zhang X. Liu X. Liu X. Huang X. (2021). Comparative efficacy of Chinese herbal injections for treating severe pneumonia: a systematic review and bayesian network meta-analysis of randomized controlled trials. Front. Pharmacol. 12, 743486. 10.3389/fphar.2021.743486 35082663 PMC8784988

[B95] Peng F. Zou M. Z. Chen H. Q. Zhu L. X. (2015). Clinical outcomes of Tanreqing combined with azithromycin for treating Mycoplasma pneumonia in children. Henan Med. Res. 24 (11), 28–29. (in Chinese). 10.3969/j.issn.1004-437X.2015.11.013

[B96] Peng L. Q. (2013). The effect of azithromycin combined with Reduning injection in pediatric Mycoplasma pneumonia. Jilin Med. J. 34 (36), 7644–7645. (in Chinese). 10.3969/j.issn.1004-0412.2013.36.057

[B97] Qi B. (2016). Efficacy on Mycoplasma pneumonia treated with Tanreqing injection and Azithromycin in 43 cases of children. J. Med. Forum 37 (10), 60–61. (in Chinese).

[B98] Qin Z. X. (2018). Clinical study on the treatment of Mycoplasmal pneumonia in children with Xi-Yan-ping injection. master Henan Univ. Traditional Chin. Med.

[B99] Quan Y. P. (2016). Efficacy analysis of Tanreqing and azithromycin in pediatric Mycoplasma pneumonia. Yiyao Qianyan 6 (2), 106–107. (in Chinese). 10.3969/j.issn.2095-1752.2016.02.089

[B100] Ruan L. Y. Ji Y. N. Zhang Y. X. Zhang C. C. Zhang G. Y. Feng S. F. (2017). Clinical observation of 80 cases of Mycoplasma pneumoniae pneumonia. Chin. J. Pract. Pediatr. 32 (12), 956–958. (in Chinese). 10.19538/j.ek2017120618

[B101] Sánchez-Vargas F. M. Gómez-Duarte O. G. (2008). Mycoplasma pneumoniae-an emerging extra-pulmonary pathogen. Clin. Microbiol. Infect. 14 (2), 105–117. 10.1111/j.1469-0691.2007.01834.x 17949442

[B102] Shah S. S. (2019). Mycoplasma pneumoniae as a cause of community-acquired pneumonia in children. Clin. Infect. Dis. 68 (1), 13–14. 10.1093/cid/ciy421 29788200

[B103] Shang Y. X. Shen C. Stub T. Zhu S. J. Qiao S. Y. Li Y. Q. (2022). Adverse effects of andrographolide derivative medications compared to the safe use of herbal preparations of Andrographis paniculata: results of a systematic review and meta-analysis of clinical studies. Front. Pharmacol. 13, 773282. 10.3389/fphar.2022.773282 35153776 PMC8831758

[B104] Sheng A. M. (2014). Tanreqing injection combined with azithromycin for pediatric Mycoplasma pneumoniae pneumonia. Chin. J. Pract. Med. (12), 117–118. (in Chinese). 10.3760/cma.j.issn.1674-4756.2014.12.062

[B105] Shi B. Q. Huang J. H. Liu C. X. An Z. J. (2015). Clinical research of Reduning injection combined with azithromycin in treating children with Mycoplasma pneumonia. Med. J. Chin. People's Armed Police Force 5, 479–481. (in Chinese). 10.14010/j.cnki.wjyx.2015.05.016

[B106] Shi Y. F. (2009). Treatment of 40 cases of Mycoplasma pneumoniae in children with integrated Chinese and Western medicine. Chin. Pediatr. Integr. Traditional West. Med. 1 (04), 364–365. (in Chinese). 10.3969/j.issn.1674-3865.2009.04.031

[B107] Shu K. P. Yin W. X. Wu H. Y. (2021). Effectiveness of Reduning injection combined with azithromycin in sequential therapy for Mycoplasma pneumonia. World Lest Med. Inf. Abstr. 21 (72), 213–214. (in Chinese). 10.3969/j.issn.1671-3141.2021.72.103

[B108] Shu Y. F. Liu Y. (2016). Analysis of superiority of azithromycin combined with Xiyanping in children with Mycoplasma pneumoniae pneumonia. Electron. J. Clin. Med. Literature 3 (55), 11005.

[B109] Song X. P. (2011). Analysis of the efficacy of Yanhuning and azithromycin in pediatric Mycoplasma pneumonia. Chin. Med. Innov. 8 (18), 76–77. (in Chinese). 10.3969/j.issn.1674-4985.2011.18.044

[B110] Song Y. H. Yang H. Y. (2014). The effect of Xixinnao combined with azithromycin in 50 cases of pediatric Mycoplasma pneumonia. Shandong Med. J. 35, 29. (in Chinese). 10.3969/j.issn.1002-266X.2004.35.059

[B111] Sterne J. A. C. Savović J. Page M. J. Elbers R. G. Blencowe N. S. Boutron I. (2019). RoB 2: a revised tool for assessing risk of bias in randomised trials. Bmj 366, l4898. 10.1136/bmj.l4898 31462531

[B112] Su W. (2011). Clinical observation of yanhuning combined with azithromycin in pediatric Mycoplasma pneumonia. Mod. J. Integr. Traditional Chin. West. Med. 20 (05), 542–543. (in Chinese). 10.3969/j.issn.1008-8849.2011.05.011

[B113] Sun L. J. (2005). The effect of azithromycin plus Chuanhuning in 44 cases of Mycoplasma pneumonia in children. Med. J. Commun. 06, 676+678. (in Chinese). 10.3969/j.issn.1006-2440.2005.06.081

[B114] Tan D. D. (2017a). Efficacy and safety analysis of azithromycin combined with Reduning injection in Mycoplasma pneumoniae infection. Health World 7 (27), 6–7. (in Chinese). 10.3969/j.issn.2095-1752.2017.27.014

[B115] Tan X. (2017b). Comparison of the efficacy of Tanreqing and azithromycin in pediatric Mycoplasma pneumonia. Yiyao Qianyan 7 (16), 313–314. (in Chinese). 10.3969/j.issn.2095-1752.2017.16.264

[B116] Tang X. M. (2015). Clinical observation of azithromycin combined with Xiyanping in pediatric Mycoplasma pneumonia. Clin. Res. Traditional Chin. Med. 7 (32), 123–124. (in Chinese). 10.3969/j.issn.1674-7860.2015.32.072

[B117] Tao B. T. (2017). Efficacy of Reduning and azithromycin in pediatric Mycoplasma pneumonia and its effect on serum inflammatory factors. J. North Pharm. 14 (01), 98–99. (in Chinese). 10.3969/j.issn.1672-8351.2017.01.086

[B118] Vester B. Douthwaite S. (2001). Macrolide resistance conferred by base substitutions in 23S rRNA. Antimicrob. Agents Chemother. 45 (1), 1–12. 10.1128/aac.45.1.1-12.2001 11120937 PMC90232

[B119] Waites K. B. Talkington D. F. (2004). Mycoplasma pneumoniae and its role as a human pathogen. Clin. Microbiol. Rev. 17 (4), 697–728. table of contents. 10.1128/cmr.17.4.697-728.2004 15489344 PMC523564

[B120] Waites K. B. Xiao L. Liu Y. Balish M. F. Atkinson T. P. (2017). Mycoplasma pneumoniae from the respiratory tract and beyond. Clin. Microbiol. Rev. 30 (3), 747–809. 10.1128/cmr.00114-16 28539503 PMC5475226

[B121] Wang A. M. Han Y. Li H. Chen H. T. (2013). Efficacy of tanreqing injection in adjuvant treatment of Mycoplasma pneumonia in children. China Pharm. 16 (02), 263–265. (in Chinese). 10.3969/j.issn.1008-049X.2013.02.038

[B122] Wang F. G. (2019). Clinical effect of Xiyanping and azithromycin in Mycoplasma pneumonia and change of IL-8 and TNF- α. Clin. Res. 27 (06), 143–145. (in Chinese).

[B123] Wang H. Y. (2013a). Efficacy and mechanism of azithromycin in mycoplasma pneumonia in children. Mod. J. Integr. Traditional Chin. West. Med. 22 (20), 2221–2223. (in Chinese). 10.3969/j.issn.1008-8849.2013.20.024

[B124] Wang L. (2020a). Efficacy of Xiyanping injection and azithromycin in pediatric Mycoplasma pneumonia. Chin. J. Mod. Drug Appl. 14 (08), 153–154. (in Chinese). 10.14164/j.cnki.cn11-5581/r.2020.08.071

[B125] Wang L. Wu C. Q. (2013). Efficacy analysis of Tanreqing injection and azithromycin in pediatric Mycoplasma pneumonia. J. Pediatr. Pharm. 19 (10), 28–30. (in Chinese).

[B126] Wang L. L. Du L. L. Cheng S. (2014). Clinical efficacy of azithromycin combined with Reduning in treatment of Mycoplasma pneumoniae infection in children and its influence on serum inflammatory factors. Chin. J. Nosocomiology 24 (4), 863–865. (in Chinese). 10.11816/cn.ni.2014-135162

[B127] Wang P. H. (2013b). Clinical analysis of Mycoplasma pneumonia in children. Yiyao Qianyan (36), 192–193. (in Chinese). 10.3969/j.issn.2095-1752.2013.36.178

[B128] Wang W. (2016). The clinical value of combining azithromycin with Xiyanping for treating Mycoplasma pneumonia in children. China Contin. Med. Educ. 8 (11), 222–223. (in Chinese). 10.3969/j.issn.1674-9308.2016.11.151

[B129] Wang X. Q. (2018a). Clinical effect of Qingkailing combined with azithromycin in treating pediatric Mycoplasma pneumonia. Mod. Diagnosis Treat. 29 (14), 2229–2230. (in Chinese).

[B130] Wang X. Y. (2020b). Application and pharmaceutical care of traditional Chinese medicine injections recommended by guideline in treatment of COVID-19. Chin. J. Hosp. Pharm. 40(08)**,** 847–851. 10.13286/j.1001-5213.2020.08.02

[B131] Wang Y. He Y. (2018). Comparison of the effects of azithromycin combined with Tanreqing injection and azithromycin alone in children with Mycoplasma pneumonia. Med. Diet Health (1), 21–32. (in Chinese).

[B132] Wang Y. Qiu S. Yang G. Song L. Su W. Xu Y. (2012). An outbreak of Mycoplasma pneumoniae caused by a macrolide-resistant isolate in a nursery school in China. Antimicrob. Agents Chemother. 56 (7), 3748–3752. 10.1128/aac.00142-12 22585213 PMC3393466

[B133] Wang Y. Wang Z. J. (2022). Efficacy of Xiyanping combined with azithromycin sequential therapy in the treatment of Mycoplasma pneumoniae pneumonia in children and its effects on microinflammatory status and immune indexes. Med. Sci. J. Central South China 50(05)**,** 740–743. (in Chinese). 10.15972/j.cnki.43-1509/r.2022.05.029

[B134] Wang Y. N. (2018b). Clinical observation of Tanreqing combined with azithromycin for pediatric Mycoplasma pneumonia. J. North Pharm. 15 (04), 19–20. (in Chinese). 10.3969/j.issn.1672-8351.2018.04.013 36519792

[B135] Wang Y. P. Yu W. Z. Liu X. M. (2016a). Analysis on the therapeutic value of azithromycin combined with Tanreqing in pediatric Mycoplasma pneumonia. J. ShanDong First Med. University&ShanDong Acad. Med. Sci. 37 (12), 1407–1408. (in Chinese). 10.3969/j.issn.1004-7115.2016.12.034

[B136] Wang Y. P. Zhang X. Y. Jia X. Y. Huang X. M. (2016b). Efficacy of Xiyanping and azithromycin in pediatric Mycoplasma pneumonia. Shenzhen J. Integr. Traditional Chin. West. Med. 26 (07), 131–132. (in Chinese). 10.16458/j.cnki.1007-0893.2016.07.067

[B137] Wang Z. F. Wang Y. P. Zhang H. M. Fan Y. P. Lü C. Wang Y. Y. (2020). Thinking on clinical rational use of TCM injection in the treatment of novel coronavirus pneumonia (COVID-19). Zhonghua Yi Xue Za Zhi 100 (14), E016–E1047. 10.3760/cma.j.issn.cn112137-20200221-00388 32122113

[B138] Wei C. X. (2018a). Treatment effect of Tanreqing combined with azithromycin in pediatric Mycoplasma pneumonia. Guide China Med. 16 (31), 166–167. (in Chinese).

[B139] Wei J. H. (2018b). Clinical evaluation of combination xiyanping injection and azithromycin in treatment of mycoplasma pneumonia in children. Contemp. Med. 24 (20), 108–110. (in Chinese). 10.3969/j.issn.1009-4393.2018.20.046

[B140] Wei J. X. Chao Y. L. Wei Y. C. Yi J. C. Chun Y. L. Fei F. Y. (2023). The pulmonary biopharmaceutics and anti-inflammatory effects after intratracheal and intravenous administration of Re-Du-Ning injection. Biomed. Pharmacother. 160, 114335. 10.1016/j.biopha.2023.114335 36724641

[B141] Wen R. Q. (2014). Yanhuning combined with azithromycin in 40 children with Mycoplasma pneumonia. Guid. J. Traditional Chin. Med. Pharmacol. 20 (08), 115–116. (in Chinese). 10.13862/j.cnki.cn43-1446/r.2014.08.044

[B142] Wu A. W. (2013). Clinical curative effect of tanreqing injection in the treatment of infantile mycoplasma pneumonia. Chin. Foreign Med. Res. 11 (02), 1–2. (in Chinese). 10.14033/j.cnki.cfmr.2013.02.097

[B143] Wu J. R. Zhang X. M. Zhang B. (2015). Potassium Dehydroandrographolide Succinate Injection for the treatment of child epidemic parotitis: a systematic review and meta-analysis. Chin. J. Integr. Med. 21 (11), 866–873. 10.1007/s11655-014-1895-2 25491538

[B144] Xi l. (2010). Efficacy of Yanhuning combined with azithromycin in pediatric Mycoplasma pneumonia. Pract. Clin. J. Integr. Traditional Chin. West. Med. 10 (04), 44–45. (in Chinese). 10.3969/j.issn.1671-4040.2010.04.030

[B145] Xia Y. H. Pan T. L. (2016). The Reduning combined with azithromycin treatment of Mycoplasma pneumonia in children and safety evaluatio. North. Pharm. 13 (11), 137–138. (in Chinese).

[B146] Xiao W. J. (2011). Clinical observation of traditional Chinese medicine Tanreqing combined with azithromycin in pediatric Mycoplasma pneumonia. J. Guiyang Coll. Traditional Chin. Med. 33 (06), 74–75. (in Chinese). 10.3969/j.issn.1002-1108.2011.06.37

[B147] Xiaomeng Z. Jiarui W. Bing Z. Ling D. (2015). Potassium dehydroandrographolide succinate injection for treat-ment of infantile pneumonia: a systematic review and Meta-analysis. J. Tradit. Chin. Med. 35 (2), 125–133. 10.1016/s0254-6272(15)30019-4 25975044

[B148] Xiong R. Y. Peng C. (2011). The curative effect of azithromycin combined with Tanreqing in pediatric treatment of Mycoplasma pneumonia. China Mod. Med. 18 (13), 57–60. (in Chinese). 10.3969/j.issn.1674-4721.2011.13.030

[B149] Xu B. H. (2020). Clinical observation of reduning injection combined with azithromycin in treatment of mycoplasma pneumonia in children. J. Pract. Traditional Chin. Intern. Med. 34 (10), 94–96. (in Chinese). 10.13729/j.issn.1671-7813.Z20190291

[B150] Xu D. Y. (2011). Comparative analysis of Tanreqing combined with azithromycin for pediatric Mycoplasma pneumonia. Chin. J. Prev. Control Chronic Dis. 19 (06), 631–632. (in Chinese).

[B151] Xu H. Y. (2017). Analysis of clinical efficacy and safety of Tanreqing injection combined with azithromycin in pediatric Mycoplasma pneumonia. Chin. baby (11), 119–120. (in Chinese). 10.3969/j.issn.1671-2242.2017.11.112

[B152] Xu X. R. Zhang H. Huang H. Z. Ling Z. B. Deng G. X. (2020). Efficacy of Xiyanping combination and azithromycin in sequential treatment of Mycoplasma pneumonia. Health (1), 137–138. (in Chinese).

[B153] Yan J. Yi J. b. Xiao X. H. (2010). Evaluation of the curative effect of azithromycin and Tanreqing injection in the treatment of Mycoplasma pneumonia in children. Prog. Mod. Biomed. 10 (12), 2339–2341+2345. (in Chinese). 10.13241/j.cnki.pmb.2010.12.041

[B154] Yang C. Song C. Wang Y. Zhou W. Zheng W. Zhou H. (2022). Re-Du-Ning injection ameliorates radiation-induced pneumonitis and fibrosis by inhibiting AIM2 inflammasome and epithelial-mesenchymal transition. Phytomedicine 102, 154184. 10.1016/j.phymed.2022.154184 35665679

[B155] Yang J. (2014). Efficacy and safety evaluation of xiyanping adjunctive azithromycin therapy in treatment of children mycoplasma pneumonia. China Med. 9 (12), 1746–1748. (in Chinese). 10.3760/cma.j.issn.1673-4777.2014.12.006

[B156] Yang S. Liu X. Wang H. Wang H. Sun D. Han Y. (2024). Wuhu decoction combined with azithromycin for treatment of Mycoplasma pneumoniae pneumonia in Asian children: a systematic review and meta analysis of randomized controlled trials. Front. Pharmacol. 15, 1329516. 10.3389/fphar.2024.1329516 38633618 PMC11021718

[B157] Yao L. (2011). Clinical efficacy of Xixinnao injection in treating pediatric Mycoplasma pneumonia. China Mod. Med. 18 (16), 66–67. (in Chinese). 10.3969/j.issn.1674-4721.2011.16.038

[B158] Yi Y. Q. (2015). Tanreqing injection combined with azithromycin treated Mycoplasma pneumoniae in 32 children. Med. Inf. 18, 296. (in Chinese). 10.3969/j.issn.1006-1959.2015.18.447

[B159] You F. Y. (2020). Research on the sequential effects of azithromycin and Tanreqing injection in treating Mycoplasma pneumonia in children. Smart Healthc. 6 (30), 192–193. (in Chinese). 10.19335/j.cnki.2096-1219.2020.30.090

[B160] Yu Y. F. (2022). Impact of Reduning and azithromycin treatment on clinical efficacy, symptom scores, immune function, and inflammatory factors in children with Mycoplasma pneumonia. Chin. health care 40 (9), 62–65. (in Chinese).

[B161] Yuan B. L. Sun Y. M. (2011). The combination of azithromycin and Xiyanping in 64 cases of pediatric mycoplasma pneumonia. Shanxi Med. J. 40 (05), 482–483. (in Chinese). 10.3969/j.issn.0253-9926.2011.10.048

[B162] Yuan F. Q. (2021). Clinical effect of Tanreqing injection for adjuvant treatment of Mycoplasma pneumoniae in children. Clin. Nurs. Res. 30 (50), 55–56. (in Chinese).

[B163] Zhang C. L. (2020). Study on the application value of Xi Xinnao injection for RMPP in children. J. Qiannan Med. Coll. Natl. 33 (02), 119–122. (in Chinese).

[B164] Zhang F. Gao Z. X. (2018). Efficacy and safety of reduning combined with azithromycin in the treatment of mycoplasma pneumoniae pneumonia in children. Med. Recapitulate 24 (17), 3533–3536. (in Chinese). 10.3969/j.issn.1006-2084.2018.17.041

[B165] Zhang H. X. (2017a). Clinical effect of sequential therapy in children with Mycoplasma pneumonia. Chin. J. Med. Device 30 (11), 11–12. (in Chinese). 10.3969/j.issn.1002-2376.2017.11.007

[B166] Zhang J. Zhang S. Tian L. Ai Z. H. (2016). Efficacy of Tanqing injection in Mycoplasma pneumonia and its effect on serum leptin, hs-CRP, IL-8 and IL-18. Mod. J. Integr. Traditional Chin. West. Med. 25 (17), 1892–1894. (in Chinese).

[B167] Zhang L. R. (2023). Efficacy of sequential therapy with Tanreqing injection and azithromycin in pediatric Mycoplasma pneumonia. Health Care 27 (3), 284–285. (in Chinese). 10.3969/j.issn.1008-0430.2023.03.114

[B168] Zhang P. (2022). Clinical effect of Tanreqing injection combined with azithromycin in sequential treatment of pediatric Mycoplasma pneumoniae pneumonia. Diabetes world 19 (3), 54–55. (in Chinese).

[B169] Zhang Q. (2011). Efficacy of azithromycin combined with Tanreqing in pediatric Mycoplasma pneumonia. Med. Forum 15 (34), 1113–1114. (in Chinese). 10.3969/j.issn.1672-1721.2011.34.018

[B170] Zhang Q. Z. Li B. F. Zhang Y. (2011). Effect of Tanreqing combined with azithromycin in pediatric Mycoplasma pneumonia. Guide China Med. 9 (26), 315–316. (in Chinese). 10.15912/j.cnki.gocm.2011.26.240

[B171] Zhang W. W. (2021). Investigate the effects of azithromycin combined with Tanreqing on clinical symptoms, inflammatory markers, and immune function in pediatric Mycoplasma pneumonia. Chin. J. Mod. Drug Appl. 15 (05), 169–171. (in Chinese). 10.14164/j.cnki.cn11-5581/r.2021.05.068

[B172] Zhang X. Shi Z. X. (2014). Analysis of the clinical effect of azithromycin combined with Yanhuning inflammatory treatment in pediatric Mycoplasma pneumonia. China Mod. Med. 21 (05), 73–74+77. (in Chinese).

[B173] Zhang X. Y. Lv L. Zhou Y. L. Xie L. D. Xu Q. Zou X. F. (2021a). Efficacy and safety of Xiyanping injection in the treatment of COVID-19: a multicenter, prospective, open-label and randomized controlled trial. Phytother. Res. 35 (8), 4401–4410. 10.1002/ptr.7141 33979464 PMC8242486

[B174] Zhang Y. (2017b). Effect of azithromycin combined with Reduning injection in children with Mycoplasma pneumoniae infection. World Latest Med. Inf. 17 (84), 75+80. (in Chinese).

[B175] Zhang Y. C. (2014). Analysis of the effect of azithromycin combined with Xixinnao injection in pediatric Mycoplasma pneumonia. Henan Med. Res. 23 (10), 52–53. (in Chinese). 10.3969/j.issn.1004-437X2014.10.028

[B176] Zhang Y. M. (2016). Clinical effect of Tanreqing injection combined with azithromycin in children with Mycoplasma pneumoniae pneumonia. Mod. Diagnosis and Treatmen 27 (17), 3172+3174. (in Chinese).

[B177] Zhang Y. X. Wang Z. J. Ruan L. Y. Zhang C. C. Zhang G. Y. Han B. (2021b). Expressions of serum hs-CRP, PCT and T cell subsets in children with mycoplasma pneumonia and clinical efficacy of andrographolide sulfonate intervention. Chin. ARCHIVES TRADITIONAL Chin. Med. 39 (04), 255–258. (in Chinese). 10.13193/j.issn.1673-7717.2021.04.065

[B178] Zhang Y. Y. (2012). Efficacy analysis of Tanreqing injection for treating Mycoplasma pneumonia in children. J. Hebei North Univ. Sci. Ed. 28 (04), 96–97. (in Chinese). 10.3969/j.issn.1673-1492.2012.04.031

[B179] Zhao C. Bai Y. Wu P. Liu X. W. Jin R. M. (2018). Xiyanping injection combined with azithromycin: effects on serum inflammatory factors and clinical outcomes in children with Mycoplasma pneumonia. Chin. J. Med. Guide 20 (04), 216–220. (in Chinese). 10.3969/j.issn.1004-437X.2018.07.050

[B180] Zhao G. X. Wang L. Yang K. Zhang H. L. Li J. S. (2022). Network Meta-analysis of heat-clearing and detoxifying Chinese medicine injections in treatment of acute exacerbation of chronic obstructive pulmonary disease. Zhongguo Zhong Yao Za Zhi 47 (10), 2788–2801. 10.19540/j.cnki.cjcmm.20220105.502 35718499

[B181] Zhao X. L. (2018). Impact of Tanreqing injection combined with azithromycin on the improvement of symptoms and adverse reactions in pediatric Mycoplasma pneumonia. Clin. Res. 26 (10), 132–133. (in Chinese). 10.3969/j.issn.1004-8650.2018.10.075

[B182] Zhen H. Z. (2014). Clinical observation of azithromycin combined with Tanreqing for pediatric mycoplasma pneumonia. China Foreign Med. Treat. 33 (09), 111–112. (in Chinese). 10.16662/j.cnki.1674-0742.2014.09.098

[B183] Zhong B. Q. Zhang S. X. Xue F. Guo Y. W. (2014). Efficacy of azithromycin combined with Tanreqing in pediatric Mycoplasma pneumonia. Med. Inf. (22), 407–408. (in Chinese). 10.3969/j.issn.1006-1959.2014.22.476

[B184] Zhou M. (2015a). Efficacy of azithromycin sequential therapy combined with Reduning pediatric Mycoplasma pneumonia. Medicial Sci. 1 (7), 151–152. (in Chinese).

[B185] Zhou Q. (2012). Azithromycin combined with potassium sodium dehydroandroan drographolide succinate versus azithromycin alone for Mycoplasma pneumonia in children. Int. Med. Health Guid. News 18 (23), 3427–3429. (in Chinese). 10.3760/cma.j.issn.1007-1245.2012.23.016

[B186] Zhou W. (2020). Effects of Reduning combined with azithromycin in treatment of Mycoplasma pneumonia in children. Med. J. Chin. People's Health 32 (22), 92–94. (in Chinese). 10.3969/j.issn.1672-0369.2020.22.039

[B187] Zhou W. Z. (2015b). Effectiveness of Tanreqing injection as an adjunctive treatment for Mycoplasma pneumonia in children and its impact on serum inflammatory cytokines. J. Clin. Med. Pract. 19 (11), 182–184. (in Chinese). 10.7619/jcmp.201511068

[B188] Zhu M. T. Luo H. M. (2018). Effect of sequential therapy with azithromycin and Reduning on Mycoplasma pneumonia in children and its impact on serum inflammatory markers. ournal Bethune Med. Sci. 16 (01), 101–103. (in Chinese). 10.16485/j.issn.2095-7858.2018.01.051

[B189] Zhu X. (2017). Clinical efficacy of Yanhuning injection adjuvant azithromycin in pediatric Mycoplasma pneumoniae. J. North Pharm. 14 (02), 112–113. (in Chinese). 10.3969/j.issn.1672-8351.2017.02.097

