# Supplementary material for: The efficacy of azithromycin combined with seven types of Chinese medicine injections in the treatment of Mycoplasma pneumoniae pneumonia in children: a systematic review and Bayesian network meta-analysis
Source: Front Pharmacol. 2024 Sep 24;15:1378445. doi: 10.3389/fphar.2024.1378445 (PMC11484089; doi:10.3389/fphar.2024.1378445)
Supplement: Supplementary file 2 [file DataSheet4.pdf]

Supplementary Material S4　The search strategies

CNKI：2148 articles 2023.5.24

（主题：肺炎支原体肺炎 ＋ 支原体肺炎 ＋ 支原体肺炎患儿 ＋ 小儿支原体肺炎 ＋ 小儿肺炎支原体感染 ＋ 儿童支原体肺炎 ＋ 儿童肺炎支原体肺炎 ＋ 儿童肺炎支原体感染（精确））AND（篇关摘：阿奇霉素（精确））AND（全文：注射液 ＋ 注射剂 ＋ 黄芪 ＋ 喜炎平 ＋ 痰热清 ＋ 丹参 ＋ 热毒宁 ＋ 炎琥宁 ＋ 清开灵 ＋ 细辛脑（精确））AND（全文：随机 + RCT（精确））

WangFang：806 articles 2023.5.24

检索表达式： 主题:(肺炎支原体肺炎 or 支原体肺炎 or 支原体肺炎患儿 or 小儿支原体肺炎 or 小儿肺炎支原体感染 or 儿童支原体肺炎 or 儿童肺炎支原体肺炎 or 儿童肺炎支原体感染) and 主题:(注射液 or 注射剂 or 黄芪 or 喜炎平 or 痰热清 or 丹参 or 热毒宁 or 炎琥宁 or 清开灵 or 细辛脑) and 全部:(阿奇霉素) and 全部:(随机 or RCT)

Database of Chinese Sci-tech Periodicals：471 articles 2023.5.24

((((((((((((((((题名或关键词=肺炎支原体肺炎 OR 题名或关键词=支原体肺炎) OR 题名或关键词=支原体肺炎患儿) OR 题名或关键词=小儿支原体肺炎 or 小儿肺炎支原体感染) OR 题名或关键词=儿童支原体肺炎 or 儿童肺炎支原体肺炎) OR 题名或关键词=儿童肺炎支原体感染) AND (((((((((任意字段=注射液 OR 任意字段=注射剂) OR 任意字段=黄芪) OR 任意字段=喜炎平) OR 任意字段=痰热清) OR 任意字段=丹参) OR 任意字段=热毒宁) OR 任意字段=炎琥宁) OR 任意字段=清开灵) OR 任意字段=细辛脑))))) AND 题名或关键词=阿奇霉素)))) AND (任意字段=随机 OR 任意字段=RCT)))))

Chinese Biomedical Literature Database：645 articles 2023.5.24

检索条件：(随机 or RCT) AND (叠氮红霉素 or 阿奇霉素) AND (注射液 or 注射剂 or 黄芪 or 喜炎平 or 痰热清 or 丹参 or 热毒宁 or 炎琥宁 or 清开灵 or 细辛脑) AND (肺炎支原体肺炎 or 支原体肺炎 or 支原体肺炎患儿 or 小儿支原体肺炎 or 小儿肺炎支原体感染 or 儿童支原体肺炎 or 儿童肺炎支原体肺炎 or 儿童肺炎支原体感染)

Pubmed: 11 articles 2023.5.24

(((Azythromycin) OR (Sumamed or Toraseptol or Vinzam or CP-62993 or Azadose or Ultreon or Zitromax or Azithromycin Dihydrate)) AND ((Injections) OR (Inject*))) AND ((Pneumonia,Mycoplasma) OR (Pneumonia,Primary Atypical or Atypical Pneumonia, Primary or Atypical Pneumonias, Primary or Pneumonias, Primary Atypical or Primary Atypical Pneumonia or Primary Atypical Pneumonias or

Mycoplasma Pneumonia or Mycoplasma Pneumonias or Pneumonias, Mycoplasma))

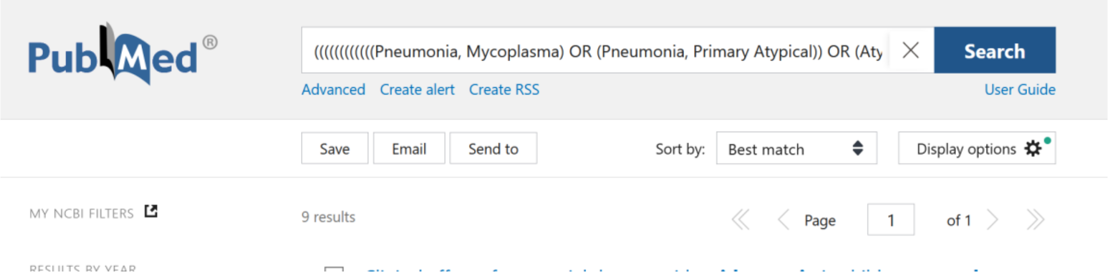

Web of Science:7 articles2023.5.24

‘Pneumonia,Primary Atypical':ta,ab or ‘Atypical Pneumonia, Primary ':ta,ab or ‘Atypical Pneumonias, Primar':ta,ab or ‘ Pneumonias, Primary Atypical':ta,ab or ‘Primary Atypical Pneumonia ':ta,ab or ‘Primary Atypical Pneumonias':ta,ab or ‘Mycoplasma Pneumonia':ta,ab or ‘Mycoplasma Pneumonias':ta,ab or ‘ Pneumonias, Mycoplasma':ta,ab

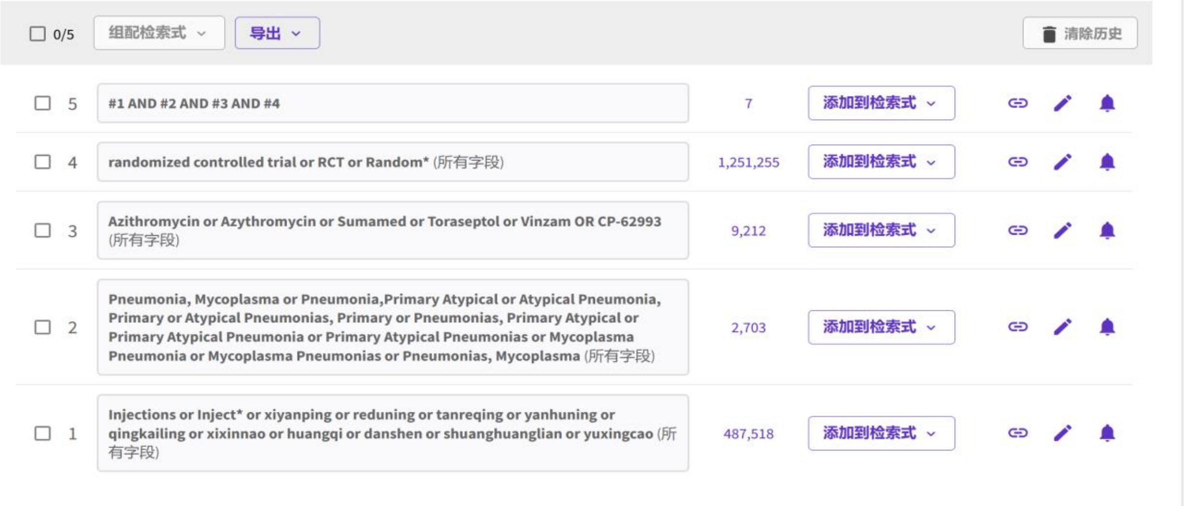

Embase:31 Articles 2023.5.24

No. Query Results Results Date

#12. #10 AND #11 31

#11. random* 2,216,387

#10. #3 AND #6 AND #9 89 20

#9. #7 OR #8 5,834

#8. 'pneumonia,primary atypical':ta,ab OR 'atypical 1,014

	pneumonia, primary':ta,ab OR 'atypical pneumonias, primar':ta,ab OR 'pneumonias, primary atypical':ta,ab OR 'primary atypical pneumonia':ta,ab OR 'primary atypical pneumonias':ta,ab OR 'mycoplasma pneumonia':ta,ab OR 'mycoplasma pneumonias':ta,ab OR 'pneumonias, mycoplasma':ta,ab	
#7.	'mycoplasma pneumonia'/exp OR 'mycoplasma pneumonia'	5,787
#6.	#4 OR #5	1,205,432
#5.	'injection'/exp OR 'injection'	838,111
#4.	'inject*'	1,197,198
#3.	#1 OR #2	58,931
#2.	'sumamed':ta,ab OR 'toraseptol':ta,ab OR 'vinzam':ta,ab OR 'cp-62993':ta,ab OR 'azadose':ta,ab OR 'ultreon':ta,ab OR 'zitromax':ta,ab OR 'azithromycin dihydrate':ta,ab	81
#1.	'mycoplasma pneumonia'/exp OR 'mycoplasma pneumonia' OR 'azithromycin'/exp OR 'azithromycin'	58,919

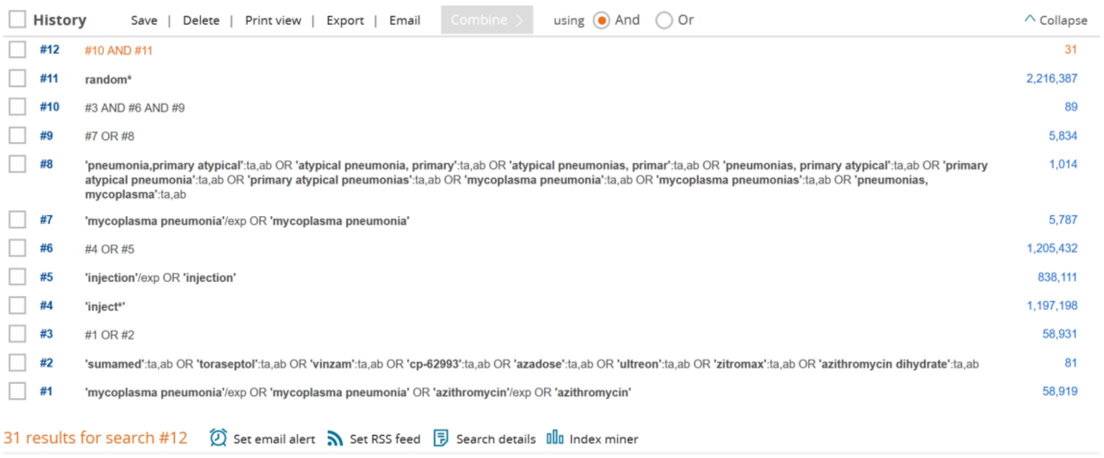

History Save | Delete | Print view | Export | Email Combine using And Or Collapse

#12	#10 AND #11	31
#11	random*	2,216,387
#10	#3 AND #6 AND #9	89
#9	#7 OR #8	5,834
#8	'pneumonia,primary atypical':ta,ab OR 'atypical pneumonia, primary':ta,ab OR 'atypical pneumonias, primar':ta,ab OR 'pneumonias, primary atypical':ta,ab OR 'primary atypical pneumonia':ta,ab OR 'primary atypical pneumonias':ta,ab OR 'mycoplasma pneumonia':ta,ab OR 'mycoplasma pneumonias':ta,ab OR 'pneumonias, mycoplasma':ta,ab	1,014
#7	'mycoplasma pneumonia'/exp OR 'mycoplasma pneumonia'	5,787
#6	#4 OR #5	1,205,432
#5	'injection'/exp OR 'injection'	838,111
#4	'inject*'	1,197,198
#3	#1 OR #2	58,931
#2	'sumamed':ta,ab OR 'toraseptol':ta,ab OR 'vinzam':ta,ab OR 'cp-62993':ta,ab OR 'azadose':ta,ab OR 'ultreon':ta,ab OR 'zitromax':ta,ab OR 'azithromycin dihydrate':ta,ab	81
#1	'mycoplasma pneumonia'/exp OR 'mycoplasma pneumonia' OR 'azithromycin'/exp OR 'azithromycin'	58,919

31 results for search #12 Set email alert Set RSS feed Search details Index miner

Cochrane Library:17 articles 2023.5.24

(Injections or Inject*) AND (Pneumonia, Mycoplasma or Pneumonia,Primary Atypical or Atypical Pneumonia) AND (Azithromycin or Azythromycin or Sumamed or Toraseptol or Vinzam OR CP-62993)
