# Supplementary material for: The efficacy of azithromycin combined with seven types of Chinese medicine injections in the treatment of Mycoplasma pneumoniae pneumonia in children: a systematic review and Bayesian network meta-analysis
Source: Front Pharmacol. 2024 Sep 24;15:1378445. doi: 10.3389/fphar.2024.1378445 (PMC11484089; doi:10.3389/fphar.2024.1378445)
Supplement: Supplementary file 3 [file DataSheet6.pdf]

Supplementary Material S6 Characteristics of the included studies

Studies Id	Treatment	Sample size	Age(Mean ±SD)	Gender(F/M)	Treatment duration	Outcomes
Ji Chunyan（2022）	Az(5～10 mg/kg/d)+XYP(5～10 mg/kg/d)	86	5.4±1.2/5.5±1.4	45/41	15 d	a,b,c,d,f
Meng Yujin (2022)	Az(10 mg/kg/d)+XYP(5 mg/kg/d)	72	5.19±1.2/5.25±1.15	37/35	1 week	a,b,c,d,f
Liu Maohua et al (2017)	Az(10 mg/kg/d)+XYP(5 mg/kg/d)	80	7.9±1.3/7.7±1.4	49/31	10 d	a,b,c,d
Luo Yuzhong (2014)	Az(10 mg/kg/d)+XYP(3 mg/kg/d)	150	8.1±1.5	85/65	_	b,c,d,f
Ning Wei (2014)	Az(10 mg/kg/d)+XYP(2 mg/kg/d)	120	5.2±1.3/5.3±1.4	74/46	＞1 week	a,b,c,d,e
Yang Jie (2014)	Az(10 mg/kg/d)+XYP(0.2～0.4 mL/kg/d)	126	5±3	73/53	2 weeks	a,b,c,d,e
Wei Jianhua (2018)	Az(10 mg/kg/d)+XYP(3 mg/kg/d)	100	5.25±1.97/5.21±1.89	46/54	8 d	a,b,c,d,e
Qin Zixing (2018)	Az(10 mg/kg/d)+XYP(0.2～0.4 ml/kg/d)	72	4.09±3.17/3.41±2.43	34/32	1 week	a,b,d,f
Wang Yuping et al (2016)	Az(10 mg/kg/d)+XYP(5 mg/kg/d)	63	4.3 ± 2.1/5.5 ± 2.5	29/34	1 week	b,c,d,e
Li Ru (2016)	Az(5～10 mg/kg/d)+XYP(0.5-0.8 ml/kg/d)	84	5.5 ± 2.4/5.8 ± 2.5	52/32	＞1 week	a,b,c,d,f
Tang Xiaoma (2015)	Az(10 mg/kg/d)+XYP(5～10 mg/kg/d)	90	7.1±2.2/7.4±2.7	57/33	2 weeks	a,b,c,d
Wang Yong et al (2022)	Az(10 mg/kg/d)+XYP(200 mg/d)	124	5.95±1. 48/5.76 ±1.34	77/47	1～2 weeks	a,f
Wang Li (2020)	Az(100 ml/d)+XYP(5～10 mg/kg/d)	100	5.2±2.2/5.0±2.0	54/46	11 d	a,b,c,d
Wang Fenggui (2019)	Az(10 mg/kg/d)+XYP(5 mg/kg/d)	100	7.6±2.1	56/44	11 d	b,c,d,e,f

**Studies Id**	**Treatment**	**Sample size**	**Age(Mean ±SD)**	**Gender(F /M)**	**Treatment duration**	**Outcomes**
Shu Yifang (2016)	Az(10 mg/kg/d)+XYP(10 mg/kg/d)	160	6.5±2.2/6.2±2.3	91/69	11～13 d	a,b,c,d
Wang Wei (2016)	Az(10 mg/kg/d)+XYP(0.2～0.4 ml/kg/d）	79	9±1.1/9±0.9	46/33	10 d	a,b,c,d
Liang Chunxia et al (2015)	Az(10 mg/kg/d)+XYP(10 mg/kg/d)	98	_	58/40	11 d	a,b,c,d,e,f
Deng Yan (2015)	Az(10 mg/kg/d)+XYP(1～3 mg/kg/d）	109	5.76±1.49	67/42	2 weeks	a,b,c,d,e,f
Wang Haiyan (2013)	Az(10 mg/kg/d)+XYP(3 mg/kg/d)	120	8.1±1.6	69/51	11 d	b,c,d,f
Jiang Shuang (2013)	Az(10 mg/kg/d)+XYP(2 mg/kg/d)	84	6.3±1.6/5.6±1.3	45/39	1～2 weeks	a,b,c,d,e
Xu Xiaorong et al (2020)	Az(10 mg/kg/d)+XYP(0.2～0.4 mL/kg/d)	80	5.2±0.7/5.4±0.7	49/31	1 week	b,c,d,e
Yuan Binli (2011)	Az(10 mg/kg/d)+XYP(10 mg/kg/d)	64	6.4±2.6/6.6±2.5	32/32	3 weeks	a,b,c,d
Wang Liling et al (2014)	Az(10 mg/kg/d)+RDN(10 mL/d)	80	6.4±1. 4 /4.5±1.3	38/42	11 d	a,f
Gao Zhaoying (2023)	Az(5 mg/kg/d)+RDN(10 mL/d)	102	6.1±4.0/5.8±3.9	51/51	2 weeks	a,b,c,d,e,f
Yu Yanfang (2022)	Az(10 mg/kg/d)+RDN(10 mg/kg/d)	120	5.36±1.2/5.32±1.26	65/55	1 week	a,f
Shu Kunpeng et al (2021)	Az(10 mg/kg/d)+RDN(0.5～0.8 mL/kg/d)	80	5.85±1.54/5.91±1.62	55/25	13 d	a,f
Xu Baohong (2020)	Az(10 mg/kg/d)+RDN(0.5 ml/kg/d)	72	5.04±2.38/4.97±2.36	38/34	2 weeks	b,c,d,f
Zhou Wei (2020)	Az(10 mg/kg/d)+RDN(0.3～0.5 mL/kg/d)	82	3.87±1.06/4.01±1.5	43/39	2 weeks	a,b,c,d
Li Hongyi et al (2018)	Az(0.2 g/d)+RDN(10 ml/d)	102	6.27±1.19/6.01±1.28	50/52	15 d	a,b,c,d,e,f
Zhang Fan (2018)	Az(10 mg/kg/d)+RDN(0.5 ml/kg/d)	80	6.4 ± 1.3 /6.5 ± 0.8	50/30	1 week	a,b,c,d,f

**Studies Id**	**Treatment**	**Sample size**	**Age(Mean ±SD)**	**Gender(F /M)**	**Treatment duration**	**Outcomes**
Shi Baoquan et al (2015)	Az(10 mg/kg/d)+RDN(0.5～0.8 ml/kg/d)	104	5.4±2.2/5.8±2.0	62/42	2 weeks	a,b,c,d,e,f
Peng Liuquan (2013)	Az(10 mg/kg/d)+RDN(10 ml/d)	218	5.62±1.13/5.64±1.15	118/100	1 week	a,b,c,d
Huang Zhaonan (2017)	Az(10 mg/kg/d)+RDN(1 ml/kg/d)	80	4. 0 ± 1. 2/ 3. 9 ± 0. 7	45/35	2 weeks	b,c,d,f
Fan Tonglian (2016)	Az(10 mg/kg/d)+RDN(10 ml/d)	68	2.83±0.68/2.66±0.53	35/33	10 d	a,b,c,d
Gao Xiuqing (2015)	Az(10 mg/kg/d)+RDN(10 ml/d)	36	5.6±1.8	19/17	1 week	a,b,c,d
Chen Ruohong et al (2022)	Az(10 mg/kg/d)+RDN(0.7 mL/kg/d)	56	5.65±3.96/5.48±3.23	32/24	2 weeks	a,f
Li Shixing (2020)	Az(10 mg/kg/d)+RDN(0.5 mL/kg/d)	44	6.1 ±2.9/5.8 ±2.9	27/17	2 weeks	a,b,c,d,e
Zhu Mantang (2018)	Az(10 mg/kg/d)+RDN(0.5 mL/kg/d)	120	6.93±1.48/6.87±1.54	62/58	3 weeks	b,c,d,f
Zhang Yuan (2017)	Az(10 mg/kg/d)+RDN(10 mL/d)	200	5.49±0.32/5.12±0.64	121/79	1 week	a,b,c,d
Hou Jianjun (2017)	Az(5～10 mg/kg/d)+RDN(0.5～0.8 ml/kg/d)	92	5.8 ± 1.5/4.1 ± 1.1	55/37	11 d	a,b,c,d,f
Tao Bingtong (2017)	Az(10 mg/kg/d)+RDN(0.3～0.5 ml/kg/d)	90	6.4±3.3/6.5±3.1	48/42	5～7 d	a,e,f
Tan Dongdong (2017)	Az(10 mg/kg/d)+RDN(0.5～0.8 ml/kg/d)	64	6.1±2.2/6.3±2.4	32/32	1～2 weeks	a,b,d
Zhou Min (2015)	Az(10 mg/kg/d)+RDN(0.6 ml/kg/d)	60	5.89±2.47/6.26±2.59	33/27	7～16 d	a,b,c,d
Gao Xibin (2014)	Az(5～10 mg/kg/d)+RDN(0.5～0.8 mL/kg/d)	80	7.3±3.29/8.2±2.91	45/35	2～3 weeks	a,b,d
Chen Yuexuan (2014)	Az(12 mg/kg/d)+RDN(0.8 mL/kg/d)	110	8.2±1.3/8.4±1.1	69/41	18 d	a,f
An Weiping et al (2012)	Az(10 mg/kg/d)+RDN(10 mL/d)	164	_	88/76	2 weeks	a,b,c,d,e

Studies Id	Treatment	Sample size	Age(Mean ±SD)	Gender(F /M)	Treatment duration	Outcomes
Liu Yimin (2011)	Az(10 mg/kg/d)+RDN(10 mL/d)	176	_	101/75	2 weeks	a,b,c,d
Du Huirong (2011)	Az(10 mg/kg/d)+RDN(0.5 ml/kg/d)	96	_	59/37	11 d	a,b,c,d
Jiang Youzhi (2016)	Az(10 mg/kg/d)+TRQ(0.3～0.5 mL/kg/d)	80	6.9±2.3/7.3±1.1	42/38	1～3 weeks	a,b,d
Wang Lin (2013)	Az(10 mg/kg/d)+TRQ(0.3～0.5 mL/kg/d)	120	7.4±3.6/7.4±3.6	61/59	10~14 d	a,b,c,d,f
Zhang Ping (2022)	Az(5～10 mg/kg/d)+TRQ(0.3～0.5 mg/kg/d)	82	7.5±0.31/7.2±0.29	51/31	2 weeks	a,b,d
Jin Yuxiu (2021)	Az(10 mg/kg/d)+TRQ(0.3～0.5 mL/kg/d)	50	5.54±1.12/5.21±1.01	29/21	7～12 d	a,f
Liu Wei (2021)	Az(5～10 mg/kg/d)+TRQ(0.3～0.5 mL/kg/d)	156	4.01±2.3/3.68±2.15	80/76	12 d	a,b,c,d,f
Gao Huina (2020)	Az(10 mg/kg/d)+TRQ(0.4 mL/kg/d)	90	3.6±1.8/3.5±1.2	48/42	10 d	a,b,c,d,f
Wang Ya (2018)	Az(0.1 mL/kg/d)+TRQ(0.3 mL/kg/d)	96	5.6±1.8 /5.8±1.7	45/51	1 week	a,b,c,d
Liu Jing (2019)	Az(10 mg/kg/d)+TRQ(0.3～0.5 mL/kg/d)	62	5.22±0.54/4.82±0.87	31/31	1 week	a,b,c,d,e
Wei Chenxia (2018)	Az(10 mg/kg/d)+TRQ(0.3～0.5 mL/kg/d)	90	8.71±2.51/8.56±2.21	35/55	2 weeks	a,b,c,d,f
Zhao Xiaoli (2018)	Az(10 mg/kg/d)+TRQ(0.5 mL/kg/d)	86	9.06±1.43/8.69±1.59	37/49	2 weeks	a,b,c,d,f
Lin Jian et al (2017)	Az(10 mg/kg/d)+TRQ(0.3～0.5 mL/kg/d)	72	6.9±2.1/ 7.1 ± 2.5	40/32	10~14 d	a,b,c,d,f
Liu Zhongyu (2017)	Az(10 mg/kg/d)+TRQ(0.3～0.5 ml/kg/d)	94	4.82±1.09/4.69±1.12	56/38	11 d	a,c,d,e
Tan Chang (2017)	Az(10 mg/kg/d)+TRQ(0.3～0.5 ml/kg/d)	86	4.26±2.77/4.68±2.43	51/35	2 weeks	a,b,d
Zhang Huixin (2017)	Az(10 mg/kg/d)+TRQ(0.3～0.5 ml/kg/d)	130	5.46±2.75/5.36±2.78	69/61	2 weeks	a,b,c,d,e

Studies Id	Treatment	Sample size	Age(Mean ±SD)	Gender(F /M)	Treatment duration	Outcomes
Chen Lixia et al (2016)	Az(10 mg/kg/d)+TRQ(5 ml/kg/d)	124	7.62±3.57/7.59±3.18	66/58	2 weeks	a,b,c,d,f
Liu Juan (2016)	Az(10 mg/kg/d)+TRQ(0.3～0.5 mg/kg/d)	68	5.13±1.72/7.98±1.63	34/34	2 weeks	a,b,c,d
Qibing (2016)	Az(10 mg/kg/d)+TRQ(0.3～0.5 mL/kg/d)	86	5.73 ± 1.06	45/41	2 weeks	a,b,c,d,e
Quan Yongping (2016)	Az(10 mg/kg/d)+TRQ(0.3～0.5 mg/kg/d)	110	5.7±1.9	61/49	2 weeks	a,b,d
Wang Yanping et al (2016)	Az(10 mg/kg/d)+TRQ(0.3～0.5 mg/kg/d)	120	2.4/2.6	66/54	2 weeks	a,b,c,d
Gao Peijun (2015)	Az(10 mg/mL/d)+TRQ(0.3～0.5 mL/kg/d)	100	7.82±1.92/8.32±1.43	54/46	1～2 weeks	a,b,c,d
Wang Pinghe (2013)	Az(10mg/kg/d) +TRQ(0.3～0.5 mL/kg/d)	90	.6±4.0/5.5±4.2	48/42	10 d	a,b,c,d
Zhang Wenwen (2021)	Az(10 mg/kg/d)+TRQ(0.3～0.5 mL/kg/d)	50	5.53±1.04/5.55±1.01	27/23	1～2 weeks	a,b,d,f
Gao Fei (2021)	Az(10 mg/kg/d)+TRQ(0.5 ml/kg/d)	126	6.13±0.85/6.27±0.92	67/59	15 d	a,f
You Fengyan (2020)	Az(10～20 mg/d)+TRQ(0.5 mL/kg/d)	56	6.0±0.6/5.5±0.5	33/23	2 weeks	a,b,c,d
Lu Zhen (2017)	Az(10 mg/kg/d)+TRQ(0.5 ml/kg/d)	60	6.19±4.7/6.85±4.62	33/27	24 d	a,b,c,d
Liu Haiying (2016)	Az(10 mg/kg/d)+TRQ(0.3～0.5 ml/kg/d)	100	6.1±1.58/6.22±1.62	56/44	2 weeks	a,b,c,d
Liang Ping (2016)	Az(10 mg/kg/d)+TRQ(0.5mL/kg/d)	104	6.6±1.0/5.8±1.1	56/48	2 weeks	a,b,c,d,e
Chen Yinghui (2016)	Az(10 mg/kg/d)+TRQ(0.5 ml/kg/d)	80	_	52/28	2 weeks	a,b,c,d,e
Deng Suqin (2015)	Az(10 mg/kg/d)+TRQ(0.3～0.5 ml/kg/d)	60	5.7±2.3/5.7±2.3	37/23	2 weeks	a,f
Li Ting (2020)	Az(10 mg/kg/d)+TRQ(0.3～0.5 ml/kg/d)	180	5.44±2.57/5.51±2.66	96/84	2 weeks	a,f

Studies Id	Treatment	Sample size	Age(Mean ±SD)	Gender(F /M)	Treatment duration	Outcomes
Zhang Liru (2023)	Az(10 mg/kg/d)+TRQ(0.3～0.5 mL/kg/d)	89	3.97±0.85/4.02±0.89	48/41	16 d	a,b,c,d,e,f
Ding Jianyong (2021)	Az(10 mg/kg/d)+TRQ(0.5 mL/kg/d)	68	5.97±1.2/5.85±1.3	37/31	15 d	a,b,c,d,f
Yuan Fengqian (2021)	Az(10 mg/kg/d)+TRQ(20 mL/d)	78	6.7±2.4/6.5±2.2	41/37	15 d	a,b,c,e,f
Xu Haiyuan (2017)	Az(10 mg/kg/d)+TRQ(0.5 mL/kg/d)	82	6.31±1.24/6.27±1.31	43/39	10 d	a,c,d,e
Wang Yani (2018)	Az(5～10 mg/kg/d)+TRQ(0.3～0.5 mL/kg/d)	70	3.6±0.9/3.4±0.8	40/30	7～10 d	a,b,c,d,f
Lu Xiaohong (2017)	Az(10 mg/kg/d)+TRQ(0.5 ml/kg/d)	120	_	65/55	2 weeks	a,b,d
Gao Jianbo (2017)	Az(15 mg/kg/d)+TRQ(10 ml/d)	146	5.37±2.38/5.38±2.37	74/72	18 d	a,b,c,d,e
Zhang Yuming (2016)	Az(10 mg/kg/d)+TRQ(0.5 ml/kg/d)	76	7.2±1.8/7.6±2.2	46/30	2 weeks	a,b,d,e
Zhang Jing et al (2016)	Az(10 mg/kg/d)+TRQ(0.3～0.5 mL/kg/d	106	6.1±2.3	68/38	2 weeks	a,b,c,d,f
Yi Yiquan (2015)	Az(10 mg/kg/d)+TRQ(0.3～0.5 mg/kg/d)	64	3.2±12.5/6.3±2.9	35/29	_	a,b,c,d
Zhou Weizhong (2015)	Az(10 mg/kg/d)+TRQ(10 mL/d)	98	6.5±1.7	47/51	16 d	a,b,c,d,f
Peng Feng et al (2015)	Az(10 mg/kg/d)+TRQ(0.3～0.5 mL/kg/d)	102	5.7±2.1/5.3±2.2	60/42	2 weeks	a,b,c,d
Hu Yanju (2015)	Az(10 mg/kg/d)+TRQ(0.3～0.5 ml/kg/d)	140	9±1.37/8±1.65	81/59	1 week	a,b,c,d
Go Lizheng (2015)	Az(10 mg/kg/d)+TRQ(0.3～0.5 mg/kg/d)	84	_	45/39	2 weeks	a,b,c,d
Cai Xiaoshu (2015)	Az(10 mg/kg/d)+TRQ(0.5～1 ml/kg/d)	108	4.31±1.22	_	2 weeks	a,b,c,d
Sheng Aimian (2014)	Az(10 mg/kg/d)+TRQ(0.5 mL/kg/d)	68	_	37/31	12 d	a,b,c,d

Studies Id	Treatment	Sample size	Age(Mean ±SD)	Gender(F/M)	Treatment duration	Outcomes
Zhong Baoquan et al (2014)	Az(11 mg/kg/d)+TRQ(0.4～0.6 ml/kg/d)	96	5.14±1.21/5.12±1.04	55/41	2 weeks	a,b,c,d
Zheng Huanzhen (2014)	Az(10 mg/kg/d)+TRQ(＜20 ml/d)	76	_	41/35	2 weeks	a,b,c,d
Liang Yuebo (2014)	Az(10 mg/kg/d)+TRQ(0.4 ml/kg/d)	128	4.5±0.7/4.3±0.5	79/49	1～2 weeks	a,b,c,d
Huang Zuwang (2014)	Az(10 mg/kg/d)+TRQ(0.3～0.5 ml/kg/d)	100	8.13±3.14/8.14±3.56	53/47	2 weeks	a,b,c,d
Hu Xiaojing (2014)	Az(10 mg/kg/d)+TRQ(0.3～0.5 ml/kg/d)	90	6.4±3.1/6.6±3.2	47/43	17 d	a,b,c,d
Wu Aiwen (2013)	Az(10 mg/kg/d)+TRQ(0.5～1 ml/kg/d)	84	1.8±0.3	52/32	2 weeks	a,b,d
Wang Aimin et al (2013)	Az(10 mg/kg/d)+TRQ(0.5 ml/kg/d)	54	_	29/25	1～2 weeks	a,b,d
Liu Jinping (2013)	Az(10 mg/kg/d)+TRQ(0.5 mL/kg/d)	64	_	29/35	2 weeks	a,b,c,d,e
Li Xiaojie (2013)	Az(10 mg/kg/d)+TRQ(0.3～0.5 ml/kg/d)	120	_	68/52	2 weeks	a,b,c,d
Jiang Hongyu et al (2013)	Az(10 mg/kg/d)+TRQ(0.3～0.5 ml/kg/d)	80	7.2±3.9	41/39	1～2 weeks	a,c,d
Bo lei (2013)	Az(10 mg/kg/d)+TRQ(0.3～0.5 ml/kg/d)	120	_	63/57	2 weeks	a,b,d,e
Zhang Yanyang (2012)	Az(10 mg/kg/d)+TRQ(0.4 ml/kg/d)	110	6.5±4.3/5.7±3.1	58/52	2 weeks	a,b,c,d
Zhang Qizhen et al (2011)	Az(10 mg/kg/d)+TRQ(0.5 ml/kg/d)	70	5.3±1.1/8.6±1.7	39/31	2 weeks	a,b,c,d
Zhang Qi (2011)	Az(10 mg/kg/d)+TRQ(5～15 ml/d)	64	_	_	10～12 d	a,b,c,d
Xu Danyan (2011)	Az(10 mg/kg/d)+TRQ(0.3～0.5 mL/kg/d)	120	7.37±3.61/8.01±2.97	59/61	2～3 weeks	a,b,c,d
Xiong Rongyan (2011)	Az(10 mg/kg/d)+TRQ(0.5 ml/kg/d)	88	4.6±1.4/4.4±1.5	49/39	2 weeks	a,b,c,d,e

Studies Id	Treatment	Sample size	Age(Mean ±SD)	Gender(F/M)	Treatment duration	Outcomes
Xiao Wenjun (2011)	Az(10 mg/kg/d)+TRQ(0.5 ml/kg/d)	100	3.86±2.7/3.90±2.9	43/57	25 d	a,b,c,d
Luo Yanmei (2011)	Az(10 mg/kg/d)+TRQ(0.5 ml/kg/d)	120	_	65/55	2 weeks	a,b,c,d
Li Lichun (2011)	Az(10 mg/kg/d)+TRQ(0.3～0.5 mL/kg/d)	88	6.7±1.5/6.8±1.3	49/39	11～13 d	a,b,c,d
Chen Chongzhi (2011)	Az(10 mg/kg/d)+TRQ(0.3 ml/kg/d)	120	_	71/49	2～3 weeks	a,b,c,d
Yanjun et al (2010)	Az(5～10 mg/kg/d)+TRQ(0.3～0.5 mL/kg/d)	124	3.25±1.24/3.47±1.38	65/59	2 weeks	a,b,c,d,f
Shi Yafeng (2009)	Az(10 mg/kg/d)+TRQ(0.5～1 ml/kg/d)	120	_	66/54	2 weeks	a,b,c,d
Cheng Shuhua (2008)	Az(10 mg/kg/d)+TRQ(0.3～0.5 ml/kg/d)	70	_	39/31	2 weeks	a,b,c,d
Li Yajun et al (2018)	Az(10 mg/kg/d)+YHN(5～10 mg/kg/d)	80	3.31±0.34/3.26 ±0.37	43/37	1～2 weeks	a,b,d
Jiang Shuangying et al (2015)	Az(10 mg/kg/d)+YHN(0.5 mg/kg/d)	90	_	_	1～2 weeks	a,b,c,d
Li Lin (2018)	Az(10 mg/kg/d)+YHN(10 mg/kg/d)	78	5.21±1.28/5.34±1.83	38/40	10 d	a,b,c
Zhu Xu (2017)	Az(10 mg/kg/d)+YHN(4～8 mg/kg/d)	106	6.42±2.11/6.91±2.07	56/50	1～3 weeks	a,b,c,d
Chang Lei (2017)	Az(6～10 mg/kg/d)+YHN(8～10 mg/kg/d)	99	8.65±1.6/7.89±1.8	67/32	3 weeks	a,b,c,d
Han Xiaoxia (2015)	Az(10 mg/kg/d)+YHN(10 mg/kg/d)	53	5.6±1.8/6.1±1.5	31/22	15 d	a,b,c,d,e
Han Fei (2019)	Az(10 mg/kg/d)+YHN(5～10 mg/kg/d)	234	3.6±0.4/3.8±0.5	128/106	1 week	a,b,c,d,e
Li Yanhong (2017)	Az(10 mg/d)+YHN(6 mg/d)	46	5.2±1.6	27/19	1 week	a,b,c,d
Liu Yanli (2017)	Az(10 mg/kg/d)+YHN(5～10 mg/kg/d)	60	7.1±2.5/7.3±2.8	31/29	2 weeks	a,b,c,d

Studies Id	Treatment	Sample size	Age(Mean ±SD)	Gender(F/M)	Treatment duration	Outcomes
Ma Shujuan (2015)	Az(10 mg/kg/d)+YHN(9～10 mg/kg/d)	126	7±1.3/7±1.6	94/32	1～2 weeks	a,c,d
Zhang Xia (2014)	Az(10 mg/kg/d)+YHN(5～10 mg/kg/d)	85	7.2±2.1/7.5±2.6	56/29	2 weeks	a,b,c,d
Wen Rongqian (2014)	Az(10 mg/kg/d)+YHN(10 mg/kg/d)	79	6.0±1.0/6.2±1.1	56/23	10 d	b,c,d,e,f
Lin Shuzhen (2014)	Az(10 mg/kg/d)+YHN(5～10 mg/kg/d)	158	_	87/71	2 weeks	a,b,d,e
Zhou Qing (2012)	Az(10 mg/kg/d)+YHN(5～10 mg/kg/d)	94	4.7±1.2/5.0±1.4	65/29	2 weeks	a,b,c,d
Li Xin (2012)	Az(10 mg/kg/d)+YHN(5～10 mg/kg/d)	138	5.8±4.2/6.1±2.7	71/67	7～13 d	a,b,c,d
Jiang Xun et al (2017)	Az(10 mg/kg/d)+YHN(5～10 mg/kg/d)	120	7.2±0.7/6.4±O.6	65/55	1～2 weeks	a,b,c,d
Su Wei (2011)	Az(10 mg/kg/d)+YHN(5～10 mg/kg/d)	116	5.86±1.32/5.81±1.30	64/52	1～2 weeks	a,b,c,d
Song Xiangping (2011)	Az(10 mg/kg/d)+YHN(8～12 mg/kg/d)	152	5.2±1.3	83/69	1 week	a,b,c,d
Liu Xiaoyang (2011)	Az(10 mg/kg/d)+YHN(0.24g/d)	96	6.5±1.0	57/39	17 d	a,b,c,d
Chen Lijun (2011)	Az(10 mg/kg/d)+YHN(5～10 mg/kg/d)	125	_	62/63	10 d	a,b,c,d
Cao Meiqin (2011)	Az(10 mg/kg/d)+YHN(5～10 mg/kg/d)	72	6.3±2.5/6.1±2.8	41/31	12 d	a,b,c,d
Xi Ling (2010)	Az(10 mg/kg/d)+YHN(5～10 mg/kg/d)	94	_	51/43	_	a,b,c,d
Hu Wei (2008)	Az(10 mg/kg/d)+YHN(5～10 mg/kg/d)	100	_	60/40	10 d	a,b,c,d
Chen Songbo et al (2007)	Az(10 mg/kg/d)+YHN(5～10 mg/kg/d)	141	_	76/65	12 d	a,b,d,e
Zhang Chunlin (2020)	Az(10 mg/kg/d)+XXN(0.5 ml/kg/d)	150	8.57 ± 1.23/8.25±1.56	74/76	_	a,b,c,d,f

Studies Id	Treatment	Sample size	Age(Mean ±SD)	Gender(F /M)	Treatment duration	Outcomes
Zhang Yongcheng (2014)	Az(10 mg/kg/d)+XXN(0.5 mg/kg/d)	60	_	45/15	1～3 weeks	a
Gao Qiuzhen (2013)	Az(10 mg/kg/d)+XXN(0.5～1 mg/kg/d)	64	_	35/29	1～4 weeks	a,b,c,d
Yao Ling (2011)	Az(10 mg/kg/d)+XXN(0.5 mg/kg/d)	50	4.7±1.3/5.1±1.9	26/24	2 weeks	a,b,c,d
Song Yonghua (2014)	Az(10 mg/kg/d)+XXN(0.5～1 mg/kg/d)	98	_	53/45	10 d	a,b,c
Meng lihua (2017)	Az(10 mg/kg/d)+XXN(5 mg/kg/d)	68	6.8±3.5/7.2±3.0	40/28	10～14 d	a,b,c,d,e
Wang Xiaoqing (2018)	Az(5～10 mg/kg/d)+QKL(1 ml/kg/d)	92	4.7±2.1/4.3±1.9	51/41	1 week	a,b,c,d,f
Meng Xingyan (2014)	Az(5～10 mg/kg/d)+QKL(1 ml/kg/d)	56	_	34/22	15 d	a,b,c,d,e
Zhao Chen et al (2018)	Az(10 mg/kg/d)+XYP(7.5 ml/kg/d)	100	4.85±0.81/4.95±0.76	52/46	1～2 weeks	b,c,d,e,f
Zhang Yunxia et al (2021)	Az(10 mg/kg/d)+XYP(7.5 mg/kg/d)	68	6.37±1.29/6.33±1.40	33/35	1～2 weeks	a,b,c,d,e,f
Wang Zhengjun et al (2017)	Az(10 mg/kg/d)+XYP(0.1 ml/kg/d)	160	8.9±2.7/9.2±3.1	78/82	1～3 weeks	a,b,c,d,e
Sun Longjun (2005)	Az(10 mg/kg/d)+CHN(10 mg/kg/d)	90	6.5±2.7	46/44	10 d	a,b,c,d
An Chenghua (2009)	Az(10 mg/kg/d)+CHN(5～10mg/kg/d)	84	_	46/38	1～2 weeks	a,b,d,e
Liu Junyan (2008)	Az(10 mg/kg/d)+CHN(20～40mg/d)	82	_	48/34	1～2 weeks	b,c,d

Notes: a, clinical effective rate; b, disappearance time of cough; c, disappearance time of pulmonary rale; d, disappearance time of fever; e, average

hospitalization time; f, disappearance time of pulmonary shadows in *X*-ray. AZ, azithromycin injection; XYP, Xiyanping injection; RDN, Reduning injection; TRQ, Tanreqing injection; YHN, Yanhuning injection; XXN, Xixinnao injection; QKL, Qingkialing injection; CHN, Chuanhuning injection.
