# Supplementary material for: The efficacy of azithromycin combined with seven types of Chinese medicine injections in the treatment of Mycoplasma pneumoniae pneumonia in children: a systematic review and Bayesian network meta-analysis
Source: Front Pharmacol. 2024 Sep 24;15:1378445. doi: 10.3389/fphar.2024.1378445 (PMC11484089; doi:10.3389/fphar.2024.1378445)
Supplement: Supplementary file 4 [file DataSheet3.pdf]

Supplementary Material S3 More details about the product information of 7 CHIs

Injection name	Raw materials	Labeled efficacy/ Contraindications	Indications	Standard of authority	Adverse drug reactions	interactions
**Reduning injection**	*Artemisiae Annuae Herba, Lonicerae Japonicae Flos, Gardznize Fructus*	Clearing heat, dispelling wind, removing toxic substance. / Should not be used during breastfeeding and pregnancy.	Cold due to exogenous wind-heat, marked by high fever, headache, body pain, cough, phlegm yellow; upper respiratory tract infection and acute bronchitis	YBZ08202005 issued by China Food and Drug Administration	Dizziness, chest congestion, xerostomia, diarrhea, nausea, vomit, pruritus, skin rash, dyspnea	Caution is advised when combining medications. This product may produce turbidity or precipitation when used with penicillins, aminoglycosides, or macrolides. If it is necessary to use other medications, flush the infusion device with 5% glucose solution or 0.9% saline (more than 50 ml), or replace the infusion device, and ensure a sufficient time interval to prevent adverse reactions.
**Tanreqing injection**	*Scutellariae Radix, Cornu gorais，Lonicerae Japonicae Flos, Forsythiae Fructus*	Clearing heat-toxin, dissipating phlegm./ Contraindicated in pregnant women and use with caution in breastfeeding women. Use with caution in elderly patients, and avoid	Phlegm heat obstruct lung syndrome, such as fever, cough, expectoration, thirst, redness of tongue, and yellow fur; acute bronchitis, acute pneumonia (early)	YBZ00912003-2007Z-2009-2012 issued by China Food and Drug Administration	Dizziness, nausea, vomit, pruritus, skin rash, fever, chest congestion, edema, phlebitis, anaphylactic shock, dyspnea	When administering an intravenous drip of 250 ml 5% xylitol solution mixed with 20 ml of Phanreqing injection, followed by intravenous push of bromhexine hydrochloride, white turbidity and flocculent matter may appear in the infusion line. Therefore, when using combined

		use in cases of liver or kidney dysfunction.				medications, it is necessary to first flush the infusion line with 5% glucose solution or 0.9% saline (more than 50 ml) or replace the infusion device, and to maintain a time interval to prevent adverse reactions caused by drug interactions.
**Xixinnao injection**	*Asarone*	Calm panting and suppress cough, expelling phlegm, sedation, spasmolysis, anticonvulsant./ Exercise caution when using in cases of severe liver or kidney impairment.	Pneumonia, bronchial asthma, chronic obstructive pulmonary disease	WS-10001-(HD-0437)-2002 issued by China Food and Drug Administration	Palpitation, chest congestion, skin rash, dizziness, nausea, vomit, anaphylactic shock	This product can potentiate the hypnotic effects of barbiturates.Combining with reserpine or chlorpromazine has a synergistic effect on the central nervous system.
**Qingkailing injection**	*Bubali Cornu*, *Baicalin*,*Margaritifera*,*Concha*, *Gardeniae Fructus*, *Isatidis*	Antipyretic,anti-inflammatory,Stimulate the mind and open the orifices./This injection is contraindicated in individuals with hypokalemia or a	Upper respiratory inflammation, viral encephalitis, hepatitis, stoke, cerebal thrombosis, tonsillitis, tracheitis and high fever	WS3-Bb-0077-95 issued by China Food and Drug Administration	Anaphylactic shock,anaphylactic shock,vomit, gastrointestinal adverse reactions ,fever,pruritus	Qingkailing injection should not be combined with gentamicin sulfate, penicillin G potassium, adrenaline, methoxamine hydrochloride, erythromycin lactobionate, dopamine, lobeline, or mephentermine sulfate.

	Radix, Lonicerae Japonicae Flos	history of hypokalemia-related periodic paralysis. It is also not to be used in neonates, infants, and pregnant women.				
**Chuanhuning injection**	*potassium dehydroandrographolide succinate*	Reducing pressure in the brain,Clearing heat-toxin, analgesic./Pregnant and breastfeeding women: This product has cytotoxic effects on placental trophoblast cells, and thus should be avoided during pregnancy. For patients with renal insufficiency, use should be avoided if the endogenous creatinine clearance rate is <30 mL • min^{-1}.	Viral pneumonia and viral upper respiratory tract infection	WS-10001-(HD-0015)-2002 issued by China Food and Drug Administration	dizziness,pruritus, skin rash, gastrointestinal adverse reactions (nausea, vomit, diarrhea), shiver, fever, chest congestion	This product must not be used in combination with acidic or alkaline drugs, or with drugs containing sodium bisulfite or sodium metabisulfite as antioxidants.

**Xiyanping injection**	*Andrographolide sulfonate*	Clearing heat-toxin, suppress cough and check dysentery./contraindicated in pregnancy.	Bronchitis, tonsillitis, bacillary dysentery	WS-10863 (ZD-0863) -2002-2011Z issued by China Food and Drug Administration	Pruritus, skin rash, palpitation, diarrhea, vomit, diarrhea	No information is available on interactions between this product and other medications.
**Yanhuning injection**	*potassium sodium dehydroandrographolide succinate*	Clearing heat-toxin, antiviral./contraindicated in pregnancy.	Viral pneumonia and viral upper respiratory tract infection	YBH06272009 issued by China Food and Drug Administration	Pruritus, skin rash, gastrointestinal adverse reactions (nausea, vomit, diarrhea), reduction of leukocyte, shiver, fever, dizziness, chest congestion, palpitation	This product should not be used in combination with acidic or alkaline medications, or with drugs containing sodium bisulfite or sodium metabisulfite as antioxidants, including vitamin B6 injection, calcium gluconate injection, aminophylline, aminoglycosides, and quinolone antibiotics.
