# Supplementary material for: The efficacy of azithromycin combined with seven types of Chinese medicine injections in the treatment of Mycoplasma pneumoniae pneumonia in children: a systematic review and Bayesian network meta-analysis
Source: Front Pharmacol. 2024 Sep 24;15:1378445. doi: 10.3389/fphar.2024.1378445 (PMC11484089; doi:10.3389/fphar.2024.1378445)
Supplement: Supplementary file 6 [file DataSheet5.pdf]

Supplementary Material S5 The definite cases of adverse events in each trial

	AZ+XYP	AZ	AZ+RDN	AZ	AZ+TRQ	AZ	AZ+YHN	AZ
digestive system	(38/1245)	(65/1230)	(63/1293)	(78/1283)	(162/3273)	(271/3207)	(31/1281)	(61/1259)
Skin rash	(12/1245)	(15/1230)	(29/1293)	(26/1283)	(7/3273)	(20/3207)	(1/1281)	(1/1259)
Dizziness/ headache	/	/	(1/1293)	(2/1283)	(3/3273)	(4/3207)	/	/
Pain at the injection site	(19/1245)	(18/1230)	(3/1293)	(3/1283)	(4/3273)	(7/3207)	(11/1281)	(9/1259)
Liver dysfunction	(2/1245)	(6/1230)	(1/1293)	(0/1283)	(0/3273)	(1/3207)	(0/1281)	(2/1259)
others	(1/1245)	(6/1230)	(15/1293)	(23/1283)	(2/3273)	(4/3207)	(0/1281)	(1/1259)
Total	(72/1245)	(110/1230)	(112/1293)	(132/1283)	(178/3273)	(307/3207)	(43/1281)	(74/1259)

Notes: AZ, azithromycin injection; XYP, Xiyanping injection; RDN, Reduning injection; TRQ, Tanreqing injection; YHN, Yanhuning injection; XXN, Xixinnao injection; QKL, Qingkialing injection; CHN, Chuanhuning injection. (X/Y), X, the definite cases of adverse events, Y, total number of patients using this injection.
